# Seasonality in risk of pandemic influenza emergence

**DOI:** 10.1371/journal.pcbi.1005749

**Published:** 2017-10-19

**Authors:** Spencer J. Fox, Joel C. Miller, Lauren Ancel Meyers

**Affiliations:** 1 Integrative Biology, The University of Texas at Austin, Austin, Texas, United States of America; 2 Mathematical Sciences, Monash University, Frankston, Victoria, Australia; 3 The Institute for Disease Modeling, Bellevue, Washington, United States of America; 4 The Santa Fe Institute, Santa Fe, New Mexico, United States of America; Duke University, UNITED STATES

## Abstract

Influenza pandemics can emerge unexpectedly and wreak global devastation. However, each of the six pandemics since 1889 emerged in the Northern Hemisphere just after the flu season, suggesting that pandemic timing may be predictable. Using a stochastic model fit to seasonal flu surveillance data from the United States, we find that seasonal flu leaves a transient wake of heterosubtypic immunity that impedes the emergence of novel flu viruses. This refractory period provides a simple explanation for not only the spring-summer timing of historical pandemics, but also early increases in pandemic severity and multiple waves of transmission. Thus, pandemic risk may be seasonal and predictable, with the accuracy of pre-pandemic and real-time risk assessments hinging on reliable seasonal influenza surveillance and precise estimates of the breadth and duration of heterosubtypic immunity.

## Author summary

Influenza pandemics emerge via genomic reassortment between circulating human and animal strains. The risk of pandemic emergence should therefore be high during the flu season, when viruses are abundant and conditions favor transmission. However, the six pandemics on record since 1889 all emerged in the Northern Hemisphere following the flu season, suggesting that other forces may predictably constrain pandemic risk. We find that seasonal influenza epidemics leave a wake of immunity that impedes pandemic emergence. This transient *refractory period* is consistent with the spring-summer emergence, multiple wave dynamics of recent pandemics, and may cause initial underestimation of the viral transmission rate. These findings may improve pre-pandemic risk assessments and real-time situational awareness, particularly as we gain greater insight into the extent of immunity.

## Introduction

Influenza pandemics have emerged regularly throughout the 20th and 21st centuries, resulting in significant morbidity and mortality [1]. In preparation for future pandemics, public health agencies have enacted measures to expedite pandemic vaccine development [2]. However, the manufacturing and distribution process is still expected to take several months, as occurred following the initial identification of the 2009 H1N1 pandemic virus [2–6]. In the interim, the primary pandemic control measures will include prophylaxis and treatment with antiviral medications and social distancing measures [2, 7, 8]. Given the potential severity of disease and rapid pace of emergence, advanced warning and early response are imperative. Thus, public health agencies have established extensive surveillance networks in humans, livestock, and wild bird populations [9–11]. While these systems are designed to identify potential pandemic threats as infections arise, researchers have also conducted mutatagenesis experiments to identify *upstream* evolutionary risks, that is, potential pathways toward human infectivity and virulence [12, 13]. However, the utility of such “gain-of-function” experiments has been disputed, particularly given the risks associated with handling highly virulent influenza viruses [14].

**Fig 1:**
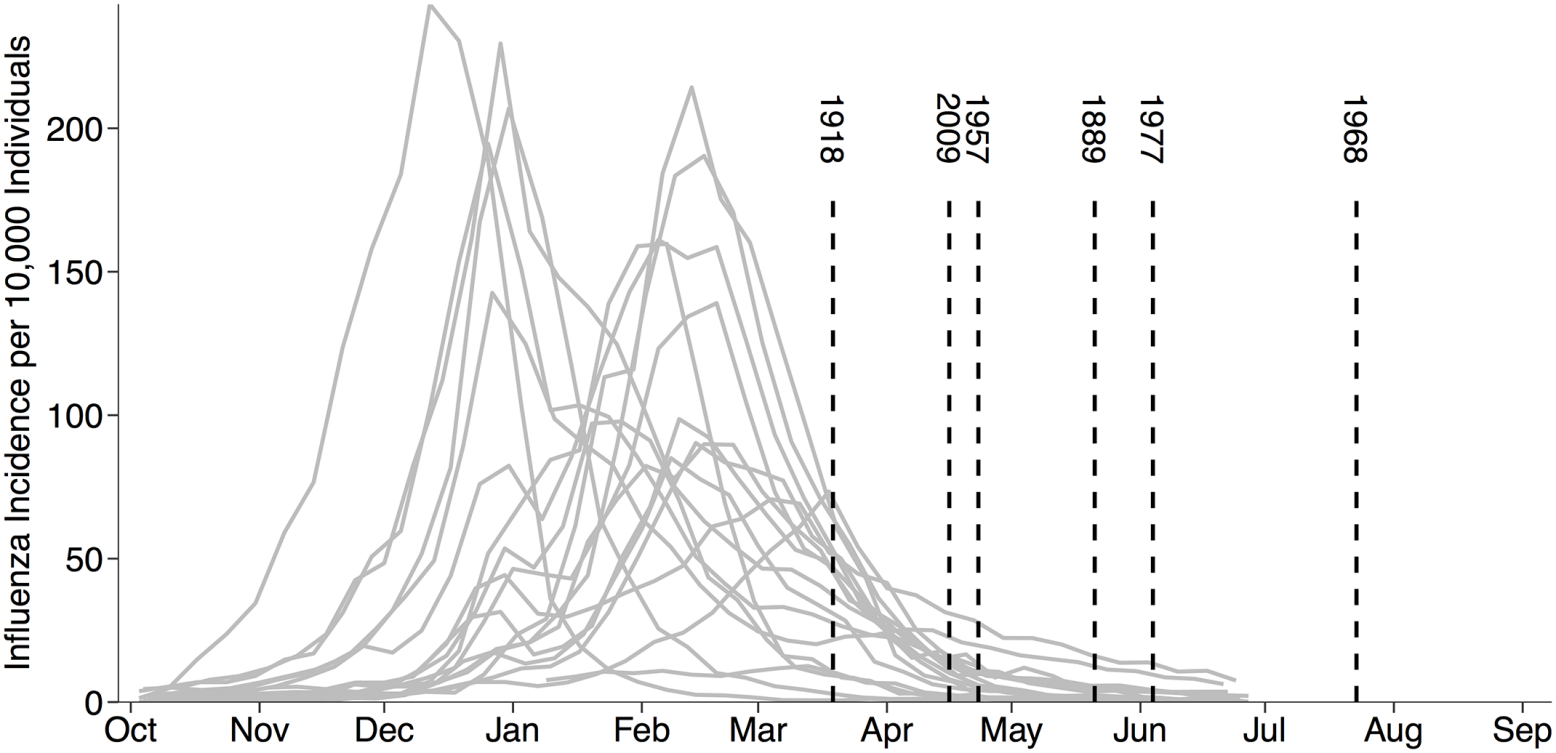
Historical pandemics emerged at the tail-end of flu seasons. Gray curves show the 1997-2015 flu seasons in the US, excluding the 2009 H1N1 pandemic, as estimated by the CDC’s ILINet surveillance system [29]. Vertical dashed lines indicate emergence week of historical pandemics in their source populations, defined as the first reported outbreak of severe influenza preceding the initial pandemic wave. These estimates were obtained from: 1889 [17], 1918 [18, 19], 1957 [20, 21], 1968 [22, 23], and 1977 [24]. To be consistent, we date the emergence of the 2009 pandemic according to the first significant outbreak preceding the initial wave, rather than the earlier outbreaks in rural Mexico that were identified only in retrospect [30].

While public health agencies cannot yet anticipate the timing and location of the next pandemic, past pandemics may provide insight into spatiotemporal trends in risk. All recent pandemics emerged in the Northern Hemisphere in the spring and summer months (Fig 1): March (1918), April (1957, 2009), May (1889, 1977), and July (1968), though the 1977 pandemic virus was highly similar to a previously circulating virus, and thus thought to have emerged via accidental release from a laboratory [15, 16]. The 1889, 1977 and 1968 pandemics produced single epidemic waves, while the 1918, 1957, and 2009 pandemics spread in two waves–a relatively short spring-summer wave followed by a more extensive fall wave [17–26]. These pandemics also varied in severity, as measured by case fatality rates, with 1918 far more severe than the others [27, 28].

The spring and summer emergence of the six recent pandemics seems more than just a coincidence (Multinomial test; *p* < 0.05), but the sample is quite small and derived from imperfect historical data. If, indeed, pandemic risk is seasonal, there are several plausible drivers. Two factors might favor pandemic emergence *during* the typical flu season. First, the socio-environmental conditions thought to promote seasonal influenza transmission (*e.g.*, humidity and school calendar) might also favor pandemic transmission during the winter months [31–33]. Second, pandemic emergence is often preceded by viral reassortment in hosts co-infected by a seasonal influenza virus and a novel virus, which should become more likely as the prevalence of seasonal flu increases [34–36]. On the other hand, transient cross-immunity from seasonal influenza infections may impede infection by novel viruses during the flu season. Together, these counterbalancing factors may produce a tight and predictable window for pandemic emergence.

Viruses of a common subtype (*e.g.* H3N2) are known to compete via *homosubtypic* immunity, producing stereotypical single branch influenza phylogenies [37–41]. However, the extent and mechanisms of competition among viruses of differing subtypes (e.g. a resident seasonal virus and a novel pandemic virus) via *heterosubtypic* immunity are not fully understood [34, 42–48]. A first childhood influenza infection may provide lifelong heterosubtypic immunity against subtypes within the same phylogenetic group (Group 1 includes H1, H2, and H5; Group 2 includes H3 and H7) [34, 37, 48–52]. In addition, any childhood or adulthood influenza infection may provide temporary, generalized heterosubtypic protection against other subtypes, lasting from a week to several months [44, 47, 53–56]. This is perhaps mediated by cells surviving influenza infection that exhibit an increased antiviral response and naturally turnover within a short period of time [53]. Immunity may not fully prevent infection, but individuals infected within this period experience reduced viral shedding, disease severity, and infection durations, likely reducing subsequent spread of the disease [45, 47, 55, 57].

Heterosubtypic immunity among influenza viruses would naturally lead to competition between subtypes, with the strength of the competition determined by the magnitude and duration of the immune response. Even if heterosubtypic immunity were short-lived, seasonal influenza may temporarily impede the emergence novel influenza subtypes. If a pandemic virus manages to emerge during this so-called refractory period, it would likely start slow and accelerate as residual immunity wanes.

Here, we characterize the impact of seasonal influenza on both the likelihood and magnitude of pandemic emergence events, mediated by transient heterosubtypic immunity following infection, and then integrate environmental constraints on flu transmission to estimate the seasonality of pandemic emergence risk. We fit two mathematical models to historical influenza data–one that assumes a homogeneous population and another that captures realistic heterogeneity in contact patterns–and simulate the introduction of novel influenza virus throughout the influenza season. We focus our analysis on the 2008-2009 seasonal epidemic, since it directly preceded the 2009 pandemic; for comparison, we also analyzed the larger 2003-2004 season (S4, S5 and S6 Figs). As expected, the risk of pandemic emergence declines in the wake of seasonal influenza, as does the effective reproduction number (early transmission rate) of any emerging pandemic. The seasonality of pandemic risk depends critically on the duration of immunity and the structure of the host population.

## Materials and methods

We developed a stochastic two-strain influenza transmission model that incorporates contact network structure, heterosubtypic immunity, and new estimates of the seasonal flu reproduction number to investigate the dynamics of pandemic emergence risk. We simulated thousands of novel pandemic virus introductions to estimate the changing probability of pandemic emergence and the *R*_eff_ upon emergence, as the flu seasons unfold.

### Two-strain influenza transmission model

We included short-term heterosubtypic immunity using a two-strain SEIPR (Susceptible-Exposed-Infectious-Protected-Recovered) network model similar to [58] (S1 Fig). All individuals are initially susceptible to both seasonal and pandemic influenza. Upon infection with one strain, individuals progress through the Exposed and Infectious classes; upon recovery, they enter a short period of complete protection from infection by the other strain, after which they regain full susceptibility to that strain. Our modeling framework does not allow simultaneous infection (co-infection) by both subtypes, as co-infection is thought to be relatively infrequent during concurrent epidemics [59]. Close sequential infections can occur in the model, as some individuals transition through the protected class almost immediately (S2 Fig). We modeled single influenza seasons, and thus assumed that recovered individuals are fully and permanently immune to their infecting strain, that there are no births or deaths, and that the network structure does not change over the course of a single simulation.

Infectious nodes infect susceptible neighbors at a per contact rate of *β*_*i*_, where *i* ∈ {seasonal, pandemic} indicates strain. We estimate *β*_seasonal_ by fitting a seasonal transmission model to recent flu season data (see data and model fitting section), and consider three transmission rate scenarios for the pandemic virus (1) equal transmissibility (*β*_pandemic_ = *β*_seasonal_), (2) lower transmissibility (*β*_pandemic_ < *β*_seasonal_), and (3) higher transmissibility (*β*_pandemic_ > *β*_seasonal_).

We assume that durations of the exposed, infectious, and recovered periods are exponentially distributed. Upon infection by either strain, individuals instantaneously enter the Exposed class, then become infectious stochastically, at rate $\eta =\frac{1}{2.26}{\text{days}}^{-1}$, recover from infection at rate $\gamma =\frac{1}{3.38}{\text{days}}^{-1}$, and leave the heterosubtypic immune period at rate $\alpha =\frac{1}{42}{\text{days}}^{-1}$, based on published estimates [53, 60]. We considered a range of heterosubtypic immune periods (S3 Fig), and herein report results based on a 42-day duration.

#### Adding seasonal forcing

We implemented seasonal forcing through a traditional humidity-forced influenza model estimating *R*_0_ through time (*R*_0_(*t*)) [61]:

$$\begin{array}{c} R_{0}\left(t\right)=\text{exp}\left(-180q\left(t\right)+\text{ln}\left(R_{0\text{max}}-R_{0\text{min}}\right)\right)+R_{0\text{min}} \end{array}$$

Where *q*(*t*) is the specific humidity at time *t*. We set *R*_0min_ = 0.8, as it is the lower bound estimate from [61], and solve for *R*_0max_ through model fitting. We used eq 3 to convert between *R*_0_ and *β*, obtaining *β*(*t*) for model fitting and simulation purposes. We used the daily average specific humidity for the United States from 2000-2016 available from NOAA [62].

### Simulation implementation

We simulated two-strain influenza epidemics using a stochastic Gillespie next-reaction algorithm built from EpiFire, a C++ network epidemic simulation library [63, 64]. We generated random contact networks with specified degree distributions using a configuration model algorithm [64]. For purposes of comparison, we assume that the homogeneous and empirical networks share the same mean degree of 〈*k*〉, with all nodes in the homogeneous network having degree exactly equal to 〈*k*〉 and the degrees in the empirical network randomly assigned according to an exponential distribution with rate $\frac{1}{\langle k\rangle }$. Based on published estimates for a large urban network, we assume 〈*k*〉 = 16 [65].

For each *scenario*—combination of contact network, pandemic introduction time, *β*_pandemic_, and immune period *α*—we ran 5,000 simulations. Each was seeded by infecting five randomly chosen individuals with the seasonal virus; at the designated introduction time, a single randomly chosen susceptible individual was infected by the pandemic virus. We terminated the simulations once no individuals remained Exposed or Infectious. For each simulation we tracked the number of nodes in each class (S1 Fig) and the *average excess degree in the susceptible portion of the network*, which is defined as follows. Consider only nodes currently susceptible to pandemic infection; call edges connecting such nodes *susceptible edges* and the number of such edges emanating from a susceptible node, the *susceptible degree* of that node. Imagine choosing a random susceptible edge and following it to one of its nodes. The average excess degree is the expected number of susceptible edges emanating from that susceptible node (other than the one along which we arrived), and is given by $\frac{\langle k_{\text{susceptible}}^{2}\rangle -\langle k_{\text{susceptible}}\rangle }{\langle k_{\text{susceptible}}\rangle }$, where 〈*k*_susceptible_〉 and $\langle k_{\text{susceptible}}^{2}\rangle$ are the average susceptible degree and average squared susceptible degree in the network. Simulation source code can be accessed at https://github.com/sjfox/EpiFire.

### Data and model fitting

To estimate the seasonal flu transmission rate, we fit a simple deterministic, network-based, ordinary differential equation (ODE) SEIR model of seasonal flu transmission to national influenza data from the United States. We chose 2008-2009 as it preceded the 2009 pandemic and 2003-2004 as a representative *large* season [29], and specifically analyzed weeks 1-15 of 2009 and week 45 of 2003 through week 3 of 2004. We estimated seasonal influenza incidence (denoted *ILI+*) throughout these periods by multiplying the CDC’s ILINet estimates of influenza activity by WHO public health lab estimates of percent positive flu tests [29], as suggested by [66].

We implemented the Volz-Miller edge-based compartmental ODE model [67–69], which is given by following equations:

$$\begin{array}{c} \begin{array}{cc} & S(t)=\psi (\theta (t))\\ & E(t)=1-S(t)-I(t)-R(t)\\ & \dot{I}\left(t\right)=\eta E\left(t\right)-\gamma I\left(t\right)\\ & \dot{R}\left(t\right)=\gamma I\left(t\right)\\ & \begin{array}{c} \dot{\phi_{I}}=\eta [\theta -\phi_{S}\left(0\right)\frac{\psi^{\prime }\left(\theta \right)}{\psi^{\prime }\left(1\right)}-\frac{\gamma (1-\theta )}{\beta }-\phi_{R}\left(0\right)]\\ \;-\left(\gamma +\beta +\eta \right)\phi_{I} \end{array}\\ & \dot{\theta }=-\beta \phi_{I} \end{array} \end{array}$$ (1)

The system can be understood by considering a test individual, *u*, which is a random individual in the network chosen at time, *t* = 0. *θ* is the overall probability that a given contact of *u* has not transmitted to *u*, and *ϕ*_*S*_, *ϕ*_*E*_, *ϕ*_*I*_, and *ϕ*_*R*_ are the probabilities that the contact has not transmitted to *u* and is currently susceptible, exposed, infectious, or recovered, respectively. *S*, *E*, *I*, and *R* denote the proportion of the population in each state, and the parameters *β*, *η*, and *γ* correspond to the per contact rate of transmission, the rate of becoming infectious upon exposure, and the recovery rate, respectively. *P*(*k*) describes the degree distribution and tells us the probability a random individual has degree *k* in the network. It follows that the average degree is given by 〈*k*〉 = ∑_*k*_ *kP*(*k*). *S*(*k*, 0) is the probability a random individual of degree *k* is initially susceptible, which leads to the probability generating function describing the proportion of susceptible individuals in the population (*ψ*) as, *ψ*(*x*) = ∑_*k*_ *S*(*k*, 0)*P*(*k*)*x*^*k*^, where *x* is the probability a given contact of *u* has not transmitted to *u*.

We match the parameters in this model to our stochastic two-strain model, including the disease progression parameters (*γ* and *η*), network structures and the initial introduction of five infections in a population of 10,000 (that is, $I\left(0\right)=\phi_{I}\left(0\right)=\frac{5}{10000}$, *ϕ*_*S*_(0) = 1 − *ϕ*_*I*_(0), *R* = *ϕ*_*R*_(0) = *ϕ*_*E*_(0) = 0, *θ*(0) = 1, and *ϕ*_*I*_ = *ϕ*_*I*_(0)). We solved the system of equations numerically using the *deSolve* package in R [70, 71].

To estimate both the per contact transmission rate, *β*_seasonal_, and time of epidemic introduction for each network, we minimized the sum of the squared errors between the 2008-2009 *ILI+* data and the incidence predictions from the ODE model, using the *optim* function in R [71]. Given that different network structures lead to different rates of epidemic growth, the flexible epidemic start time allows tighter fitting of the models to the seasonal flu incidence data. For the 2008-2009 season, we estimated the epidemic start date to be November 15th and December 18th, 2008 for the homogeneous and empirical networks, respectively [71].

### Analytic approximations of emergence probability and effective *R*_0_

We derive mean field approximations of the emergence probabilities and effective *R*_0_ using the process outlined in [72], which we outline briefly here. The generating function for the number of infected nodes in the first generation of an outbreak is given by

$$\begin{array}{c} f\left(x\right)=p_{0}+p_{1}x+\cdots +p_{j}x^{j}+\cdots \end{array}$$

where *p*_*j*_ is the probability the index case infects *j* neighbors. More specifically,

$$\begin{array}{c} p_{j}=\sum_{k=j}^{\infty }P\left(k\right)\int_{0}^{1}\mathrm{Bi}\left(k,j,T\right)P\left(T\right)dT \end{array}$$

with Bi(*k*, *j*, *T*) denoting the probability of *j* successful outcomes from *k* Bernoulli trials with probability of success equal to the transmissibility, *T*, defined by the probability distribution *P*(*T*). (The probability distribution for T is defined by the randomly drawn recovery and infectious times in the Gillespie simulation.) A node of degree *k* that has just been infected has *k* − 1 possible neighbors to infect. The probability that this node infects *j* neighbors is given by

$$\begin{array}{c} q_{j}=\frac{1}{\langle k\rangle }\sum_{k=j+1}^{\infty }kP\left(k\right)\int_{0}^{1}\mathrm{Bi}\left(k-1,j,T\right)P\left(T\right)dT \end{array}$$

Similar to *f*(*x*), *h*(*x*) = ∑ *q*_*j*_*x*^*j*^ is the generating function for the number of infections caused by a non-index case, which leads to the equation

$$\begin{array}{c} h\left(x\right)=\int_{0}^{1}\frac{P(T)}{\langle k\rangle }\sum_{k=1}^{\infty }{\left[1+T\left(x-1\right)\right]}^{k-1}kP\left(k\right)dT \end{array}$$

Ignoring finite size effects, the generating function for the number of infections *g* generations after the initial infection is *f*(*h*^*g*−1^(*x*)) where *h*^*g*−1^(*x*) denotes composition of *h* with itself *g* − 1 times. The extinction probability is the probability that eventually there are 0 infections lim_*g*→∞_ *f*(*h*^*g*−1^(0)). Setting *x*_0_ = lim_*g*→∞_ *h*^*g*−1^(0) we find that the emergence probability in a naïve network is given by

$$\begin{array}{c} P=1-f(x_{0}) \end{array}$$ (2)

It also follows that the basic reproduction number (*R*_0_) is given by

$$\begin{array}{c} R_{0}=\langle T\rangle \frac{\langle k^{2}\rangle -\langle k\rangle }{\langle k\rangle } \end{array}$$ (3)

as originally shown in [73], where 〈*T*〉 is the average transmission probability. Under our continuous-time constant-rate assumptions, this is $\langle T\rangle =\frac{\beta }{\beta +\gamma }$.

### Statistical analysis of simulated epidemics

For a given scenario, we restricted our analyses to simulations in which a seasonal epidemic actually occurred (defined as outbreaks with cumulative incidence reaching at least 5% of the population). We then estimated the pandemic emergence probability as the number of pandemic introductions that progressed into sustained outbreaks (infecting at least 5% of the population) divided by the number of simulations with seasonal epidemics of that scenario. We approximated the emergence timing of the pandemic as the first day in which the daily incidence was ≥5 individuals, as this was a good indicator for the beginning of the exponential growth phase.

Each time a pandemic successfully emerged, we estimated its *R*_eff_ by fitting the corresponding (empirical or homogeneous) single strain ODE network model (defined by equations in 1) to the simulated pandemic time series. The procedure is as described in the Data and model fitting section, with two alterations: (1) we fix the introduction time to that specified by the simulation scenario and only estimate the transmission rate, and (2) we fit the model to the *cumulative incidence* of the pandemic virus. To then obtain an *R*_0_ estimate, we plugged the estimated pandemic transmission rate and full degree distribution into eq 3.

During the *refractory period*, immunity in the population increases the transmissibility necessary for a pandemic to invade the population. We use eq 3 to estimate this changing invasion threshold; we let 〈*k*^2^〉 and 〈*k*〉 reflect the current susceptible portion of the network, set *R*_0_ = 1, and solve for *T*. For a given time point *t* and scenario, we calculated *T* for a single, prototypical simulation and divided it by the comparable threshold in a completely susceptible population. Importantly, this analysis assumes that the network susceptibility is frozen in time at the introduction time, and does not take into account subsequent epidemic and pandemic dynamics.

## Results

**Fig 2:**
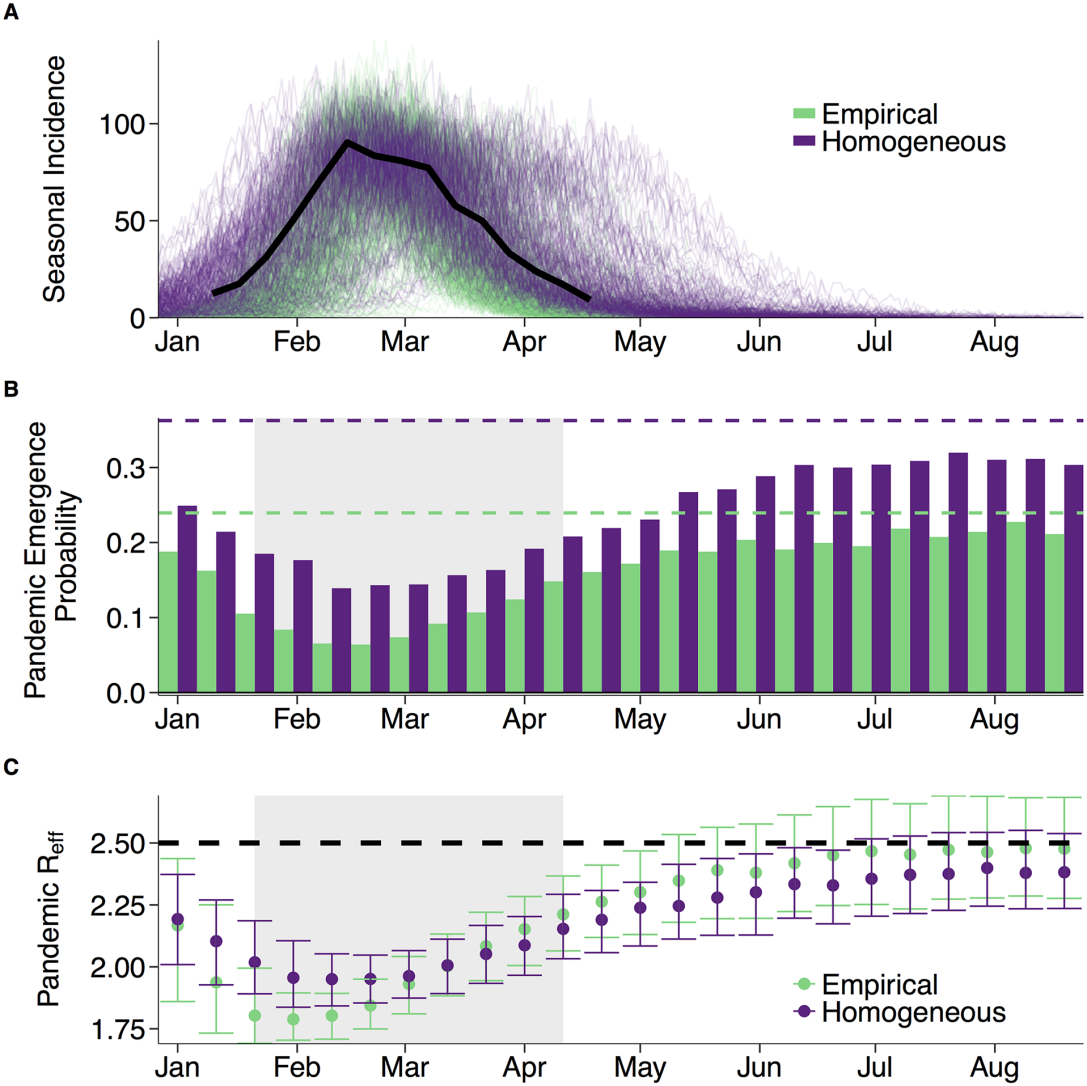
Seasonal epidemics produce a pandemic refractory period. **A**: Actual 2008-2009 epidemic curve (solid black line) and 200 stochastic simulations of seasonal epidemics for each network (green for empirical; purple for homogeneous), assuming transmission parameters estimated from 2008-2009 data. **B**: The probability of pandemic emergence upon the introduction of a single infected individual, assuming that the pandemic virus has the same transmission rate as the seasonal virus. Probability is estimated as the proportion of introductions that subsequently infected at least 5% of the overall population out of the 5,000 simulations. Horizontal dashed lines indicate the emergence probabilities in a completely susceptible population calculated with Eq 2. The pandemic refractory periods (shaded regions) are expected to occur during and immediately following the seasonal epidemic peak. **C**: Underestimation of pandemic *R*_0_. Assuming that the emerging pandemic has an *R*_0_ = 2.5 in a naïve population (dashed horizontal line), we plot the median (points) and interquartile range of the measured *R*_eff_, for each introduction time and each network. For example, if a pandemic with *R*_0_ = 2.5 emerged in March of 2009 and we did not account for population immunity, we would interpret the *R*_eff_ as the *R*_0_ and considerably underestimate the true transmission rate (*R*_0_ ≈ 2), regardless of our contact network assumptions.

We fit two network models—an empirical model and a homogeneous model (roughly equivalent to a simple mass action model)—to influenza data from the 2008-2009 season in the US (Fig 2A), and estimated reproduction numbers (*R*_0_) of 1.8 and 1.4, respectively (Analogous results for the larger 2003-2004 flu season are presented in Supplementary Material). Both estimates are consistent with prior studies [26, 74], and their discrepancy highlights a potential pitfall of simple epidemiological models. Given the observed heterogeneity in human social behavior [65], the mass action models, which assume that all individuals have identical contact rates, may underestimate epidemic potential. Using these estimates of *R*_0_, we simulate typical seasonal influenza epidemics and estimate the evolving probability of pandemic emergence. We assume an average 42-day period of complete heterosubtypic immunity upon recovery from a seasonal flu infection (S2 Fig), which corresponds to the waning of generalized immunity in a human club cell-like line [53] and lies in between other estimates [44, 47]. We provide a sensitivity analysis with respect to the duration of immunity in the supplementary information (S3 Fig).

### Pandemic refractory period

Heterosubtypic immunity is expected to reduce pandemic emergence during the seasonal epidemic, with pandemic emergence probability reaching a minimum just after the epidemic peak of the flu season and then quickly rebounding (Fig 2B). The length and intensity of this *pandemic refractory period* should increase with the duration of heterosubtypic immunity, with prolonged immunity leading to complete pandemic exclusion (S3 Fig).

The refractory period also depends on the transmissibility of the pandemic virus: the greater the transmission rate, the more readily a pandemic will emerge with or without immunological interference; the opposite is true for less transmissible viruses (S9 Fig). The refractory effect is greater in the empirical (network) model than in the homogeneous model, suggesting that mass action assumptions may lead to underestimation of viral interference and overestimation of pandemic risk. Assuming that the pandemic virus has the same intrinsic transmission rate as the seasonal virus, the probability of pandemic emergence is reduced by 73% and 62% in the empirical and homogeneous models, respectively, at the base of the refractory period, relative to comparable introductions in a completely susceptible population. Higher transmission rates lead to smaller reductions (56% and 19% respectively), while less transmissible viruses can be almost fully excluded (99% and 84% reductions respectively) (S9 Fig). The 2008-2009 influenza season was relatively mild; larger seasonal flu epidemics produce deeper refractory periods, as illustrated for the 2003-2004 influenza season (S4, S5 and S6 Figs).

### Underestimation of pandemic R_0_

For each simulated pandemic that successfully emerges, we estimate the effective *R*_0_ (*R*_eff_) of the virus, the reproduction number of the disease in a population that is not fully susceptible. Its magnitude depends on the extent of immunological interference by seasonal flu. Generally, the *R*_eff_ of the emerging pandemic virus decreases as the seasonal epidemic progresses towards its peak, bottoming out slightly before the emergence probability reaches its minimum. However, this occurs slightly earlier and more precipitously in the empirical model than in the homogeneous model (Fig 2C).

Whether or not a virus emerges depends on its intrinsic infectiousness and structure of the susceptible portion of the population. During the refractory period, the susceptible population is diminished, both in number and connectivity. At the peak of the refractory period in the empirical network, we estimate that a introduced virus must be 1.16 times more infectious (transmissible) to emerge, relative to one entering a completely susceptible population. If the seasonal epidemics preceding the 1918 and 2009 pandemics were similar in timing and magnitude to our simulated epidemics, then we estimate that their intrinsic *R*_0_’s would have been 1.08–1.20 and 1.05–1.13 times larger, respectively, than their *R*_eff_’s as the first waves emerged.

### Contact networks determine pandemic vulnerability

The different levels of pandemic risk observed in our two models stem from their underlying contact networks. To illustrate this, we use *nodes* and *edges* to represent individuals and contacts between individuals, respectively. The *degree* of a node is defined as the number of edges connecting it to other nodes. The homogeneous model assumes that all individuals have the same number of contacts; the empirical model assumes realistic variation in degree [65]. We constrained the two models to have the same total number of nodes and empirically-derived mean degree, and, consequently, the same total number of edges.

**Fig 3:**
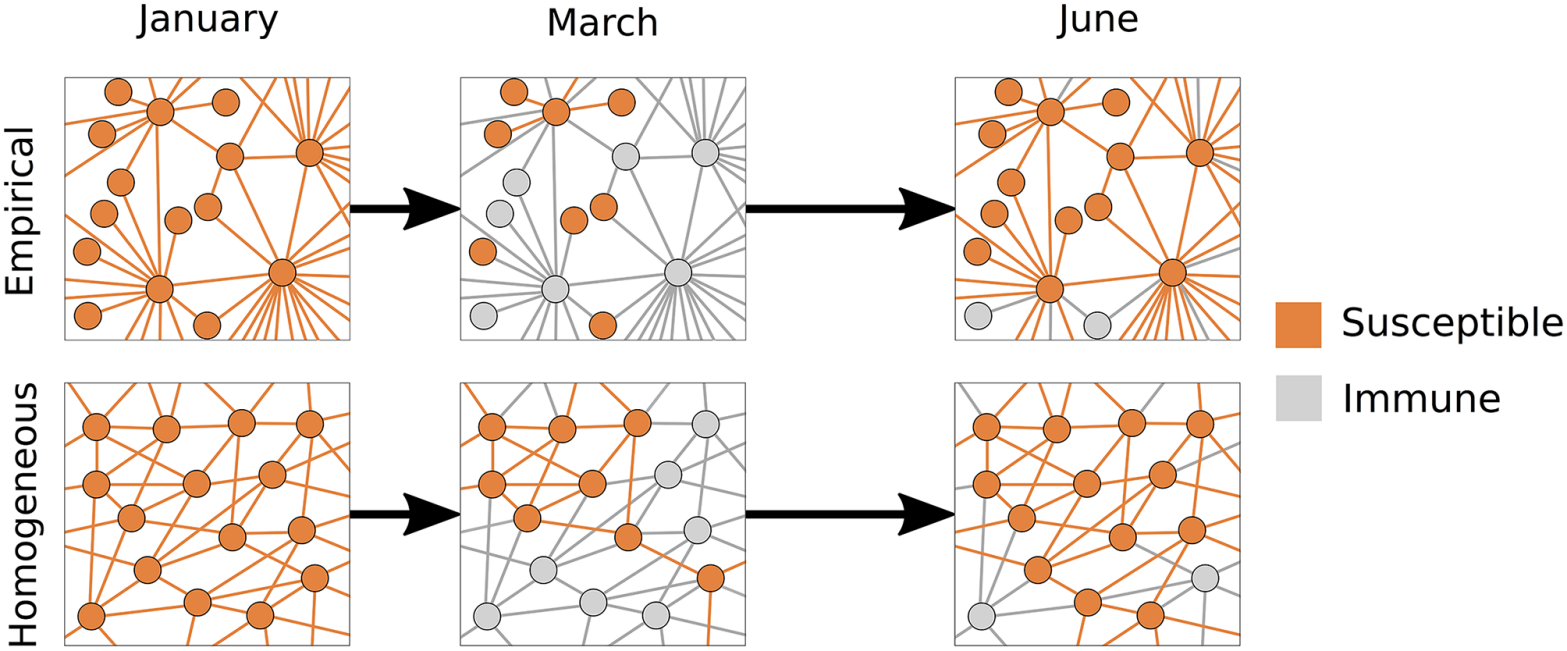
The evolving structure of the susceptible population as the flu season unfolds. For purposes of illustration, we present *caricatures* of each model through time, assuming that the average degree is 〈*k*〉 = 6 and that we repeatedly observe the same subset of each population. Orange represents individuals susceptible to infection by the pandemic virus and the contacts between them; gray indicates individuals who are currently or recently infected by the seasonal virus, and thus immune to pandemic infection. The empirical (top) and homogeneous (bottom) networks experience different structural changes in pandemic susceptibility throughout the flu season. In January, prior to the onset of flu season, both networks are fully susceptible. Just following the seasonal epidemic peak (March), both networks are at the base of their refractory period, with many nodes resistant to the pandemic virus. Even with the same number of susceptible nodes, the empirical network is more disrupted than the homogeneous network. Highly connected (hub) nodes are more vulnerable to seasonal infection than less connected nodes and, once removed by immunity, critically disconnect the susceptible portion of network. After the seasonal epidemic has subsided (June), short-term immunity has largely waned in both models, leaving them vulnerable to pandemic invasion.

The *susceptible* portion of a network is the subset of individuals that are currently susceptible to pandemic infection and any connections among them (Fig 3, orange circles and lines). (This is also known as the *residual* network [75].) The *susceptible degree* of a susceptible node is the number of contacts it has with other susceptibles. Upon infection by seasonal flu, individuals and their coincident edges leave the susceptible network, returning only when their heterospecific influenza immunity wanes. This dynamic wake of immunity depends on the underlying network structure and, importantly, determines the population’s vulnerability to pandemic emergence (Fig 3, grey nodes and edges).

As a disease spreads, the chance of a node becoming infected will depend on its degree. The more contacts a node has, the higher its exposure risk. In a homogeneous network, chains of transmission progress randomly; in a heterogeneous network, outbreaks hit the most connected nodes earliest and hardest. Consider two emerging outbreaks–one in the empirical network and another in the homogeneous network–that have reached the same cumulative incidence. Although the susceptible networks will have identical numbers of susceptible nodes, the empirical susceptible network will be much sparser (fewer edges) than the homogeneous counterpart, and will thus be more refractory to pandemic invasion (Fig 3, middle panels).

**Fig 4:**
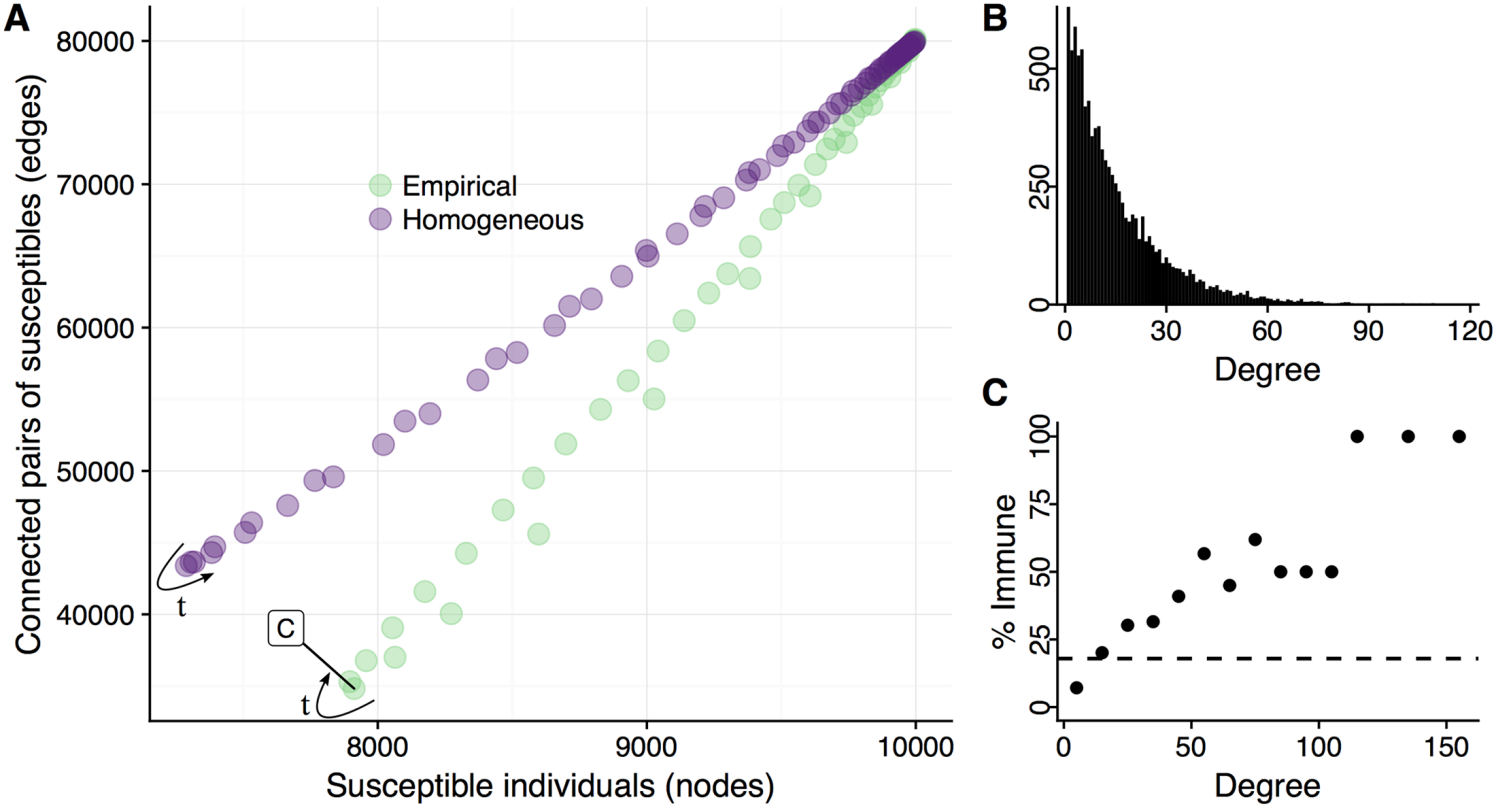
Seasonal flu disconnects the susceptible portion of a population. **A**: For a single (typical) seasonal epidemic simulation, the number of individuals susceptible to infection by a pandemic virus and the number of edges connecting two such individuals are plotted for each network (green for empirical; purple for homogeneous), with each point representing a single time point over the course of the epidemic. Arrows indicate temporal progression. For any given number of remaining susceptible individuals, the empirical model is always sparser than the homogeneous model (that is, it has fewer contacts remaining between susceptibles). **B**: The distribution of degrees (number of contacts) assumed for the empirical model. The homogeneous model assumes that all individuals have 16 contacts. **C** Snapshot of the susceptible portion of the empirical network at the base of the refractory period (at the time point indicated in panel A by the box labeled ‘C’). Points indicate the percent of the nodes that are immune to pandemic infection, across different levels of connectivity. (We bin degrees by 10; for example, the lowest bin includes individuals with 1 to 10 contacts). For comparison, the horizontal dashed line indicates the overall proportion of individuals immunized in the network at the base of the refractory period. In comparison to an individual with an average number of contacts, a highly connected individual will be more vulnerable to seasonal flu infection, and, once infected and immunized, cause greater epidemiological disruption.

In a randomly selected pair of simulations, the homogeneous network decays to an susceptible network consisting of 71% of its original nodes and 43% of its original edges, before rebounding (Fig 4A). In contrast, the empirical network maintains more nodes (78%) and fewer edges (36%) at its most refractory moment, with the high degree nodes bearing the brunt of the seasonal epidemic (Fig 4B and 4C).

### Seasonal pandemic emergence timing

**Fig 5:**
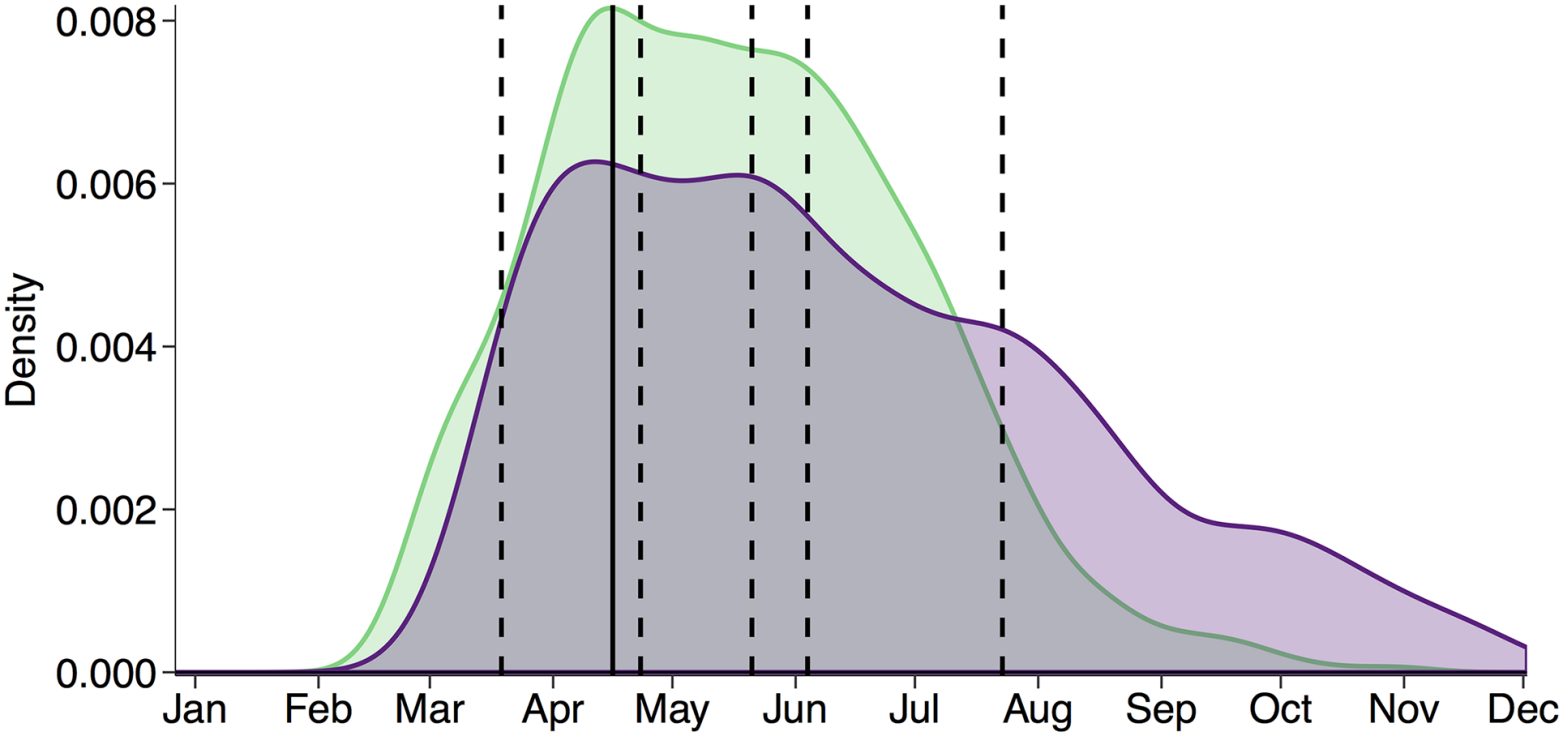
Seasonality further constrains pandemic emergence timing. Probability density for pandemic emergence timing for pandemics that emerge during the seasonal influenza epidemic for the homogeneous (purple) and empirical (green) networks. Pandemic emergence timing, the time in which the simulated pandemic begins rapid spread, is defined as the day the pandemic strain incidence reaches five or more cases. Results are for a pandemic emerging during the 2008-2009 flu season with the same transmission rate as the seasonal epidemic. Vertical lines indicate the timing of historic pandemics, with the solid line indicating the timing of the 2009 pandemic and dashed lines indicating timing of others.

The above analyses assume that pandemic emergence is constrained solely by heterosubtypic immunity, and do not consider the socio-environmental factors that shape seasonal flu dynamics. When we incorporate humidity-forced seasonality into the model, we find that pandemics are most likely to emerge soon after the seasonal epidemic peak (Fig 5), with the timing more constrained to the spring and early summer in the empirical network than in the homogeneous network. For both models, the most probable week of emergence falls within one week of the actual 2009 pandemic emergence event. To assess the consistency of our models with observed pandemics (five emerged in the spring and one emerged in the summer), we conduct multinomial tests of the model-derived probabilities of emergence across each of the four seasons. While the empirical and homogeneous models are consistent with recent history (multinomial exact test *p* = 0.53 and *p* = 0.35, respectively), the simple null model in which emergence risk is assumed to be constant throughout the year is not (*p* < 0.05). Although all historic pandemics seem consistent with the model, we note that these estimates are based on the 2008-2009 flu season and thus strictly pertain only to the 2009 pandemic. We speculate that projections from the seasons preceding each of the other historical pandemics would be similar and perhaps even better aligned with the emergence of the corresponding pandemic.

Earlier seasonal epidemics give rise to earlier risks of pandemic emergence (S6 Fig), and extending the period of pandemic introduction from just the flu season to the entire year reduces the spring/summer emergence probability and renders the model predictions inconsistent with historic pandemic timing (S7 Fig (*p* < 0.05) and S8 Fig (*p* < 0.05)).

## Discussion

The coincidental timing of recent pandemics may reflect multiple constraints, including environmental and behavioral factors that shape influenza’s transmissibility (*e.g.* humidity, school calendar, etc.), reassortment events mediated by co-infection, and immune-mediated competition between pandemic and established viruses [31–33, 44, 76]. On the one hand, we would expect pandemics to emerge during the flu season, when socio-environmental conditions are conducive to influenza transmission and co-infections are likely; on the other hand, those would be the months of greatest competitive interference. These competing effects suggest that the risk of pandemic emergence may be greatest at the tail of the flu season, when conditions are still favorable and co-infections are possible, but competition is waning. Consider the following scenario. A novel virus is produced by co-infection mediated reassortment during the heart of the influenza season. It initially stutters, hindered by widespread heterosubtypic immunity, but does not completely disappear. With each new infection, emergence is possible and increasingly probable, as heterosubtypic immunity dissipates. This is consistent with the timing of all recent pandemics (Fig 1), most of which (all but 1977) were caused by livestock-human reassortment viruses [34, 35, 76–78].

Pandemic emergence requires both the evolution of novel pandemic subtypes capable of human-to-human transmission and the ability of such new viruses to spread once they have appeared in humans. Our study focuses exclusively on the latter process, the success of new human-transmissible influenza viruses facing dynamic short-term heterosubtypic immunity resulting from seasonal influenza. Specifically, we have modeled a scenario in which potentially pandemic viruses appear (starting with a single infection) with constant probability during or following a typical Northern Hemisphere flu season. Individuals infected by seasonal flu are assumed to enjoy a short period of immunity towards other influenza subtypes, including the novel pandemic virus. Under reasonable assumptions regarding the duration of heterosubtypic immunity and human contact patterns, we characterize the changing risk of pandemic emergence throughout the flu season and find a considerable refractory period that is consistent with historical pandemic emergence events in the spring and summer months.

The rate of pandemic spread will depend on the time of emergence. Pandemics emerging during the seasonal refractory period will initially grow slowly and accelerate as residual immunity dissipates. Thus, the threat and pace of global expansion may far exceed projections based on early estimates of the viral reproduction number (*R*_0_). In our model based on the 2008-2009 flu season, we estimate that, at the peak of the refractory period, naturally occurring immunity will reduce the probability of pandemic emergence 73% and reduce the reproduction number of a successfully emerging virus by 30%. We assumed that all recovered individuals experience full protection during their short period of heterosubtypic immunity. If, for example, heterosubtypic immunity is incomplete or fails to prevent subsequent spread, the refractory effect may be diminished. Nonetheless, the qualitative results, including the timing of the refractory period and differences between the two network models should still hold.

Our comparison between homogeneous and empirical contact networks suggests that, while the refractory effect is robust, estimation of pandemic risk prior to and during emergence events will be highly sensitive to statistical assumptions regarding population structure. Several other studies have examined the impact of network structure on herd immunity following an epidemic or vaccination campaign, and similarly found that contact heterogeneity amplifies the refractory effect [75, 79–81]. Conventional models, that ignore social heterogeneity, are likely to overestimate both the emergence risk during the refractory period and the early transmission rate (*R*_0_) of an emerging pandemic virus. Given the simplicity and growing flexibility of network methods, this further supports their scientific and public health utility [67, 82, 83].

Pandemics often emerge in multiple waves [84], including a herald wave in the spring or summer and a secondary wave the following fall or winter. These wave patterns are well documented for the 1918, 1957, and 2009 pandemics [25, 85–91]. Our results provide potential insight into this phenomenon. The asynchronous forces of heterosubtypic herd immunity and suppressive off-season conditions may constrain pandemic emergence to the immediate wake of the flu season, exactly when lingering population-wide immunity is expected to dampen the initial wave of pandemic transmission. In the months following, the limited herald wave runs its course, residual seasonal immunity continues to decline, and socio-environmental conditions slowly become more conducive to flu transmission, thereby setting the stage for a major winter pandemic wave. Early estimates of pandemic *R*_0_ that do not properly account for underlying population immunity may substantially underestimate the magnitude of the second (fall or winter) pandemic wave, as the *R*_eff_ at the time of emergence may be considerably lower than *R*_0_ in a fully susceptible population. Our analysis suggests that a pandemic emerging between March and June may produce a secondary wave with an *R*_eff_ that is 4–28% larger than the initial *R*_eff_, depending on the duration of heterosubtypic immunity, the timing of emergence, and the baseline transmissibility of the virus. Recent analyses of the 1918 and 2009 pandemic waves found that the initial waves were 3.6% and 6.5% less transmissible than the secondary wave, supporting our conclusions [61, 92]. This finding is broadly consistent with published estimates for the reproduction numbers of primary and secondary pandemic waves, with the exception of the 1918 pandemic in Denmark [26, 85]. This discrepancy may be attributable to poor data or stem from local differences in the preceding flu season or population structure.

Most historic pandemic viruses were likely created by recent livestock-human reassortment events [34–36], with two possible exceptions. The 1977 pandemic was caused by a lab escapee, and the 2009 pandemic evolved from a human-derived variant that circulated in swine for years before the precipitating reassortment event, which may have occurred in livestock several months or years prior to its 2009 emergence [35]. We have assumed that pandemic introductions will be constrained to the flu season for two reasons (Fig 5). First, the chance of a livestock-human reassortment event will depend on the prevalence of flu in both humans and livestock, and thus increase as seasonal flu gains momentum. Second, flu prevalence in livestock is thought to mirror seasonal flu in humans [93, 94]. Thus, even viruses emerging directly from livestock, without a precipitating human reassortment event, may be constrained to the same months.

When we remove this assumption and introduce pandemic viruses throughout the year, the plausible emergence times start earlier in the fall, before the seasonal flu epidemic takes hold (S7 and S8 Figs). This broader emergence scenario is inconsistent with historical pandemics, given that none emerged in the fall just prior to a seasonal epidemic. While this does not prove our seasonality assumptions, it suggests that there may be factors restricting emergence events in the fall, such as the ones we have hypothesized, or that fall emergence simply has not occurred, by chance alone, across the limited number of recent pandemics. Our study was not designed to detect such seasonal constraints on pandemic emergence (rather we assume them and analyze the consequences), but leaves this as an interesting open question for future work. Interestingly, the broader emergence scenario may apply to the 1977 pandemic, which, unlike the other recent pandemics, did not emerge directly from influenza circulating in livestocks or humans, as well as to risks associated with future gain-of-function avian influenza experiments.

This approach can be readily applied to other retrospective or predictive global risk assessments, using seasonal flu surveillance data at the relevant geographic and temporal scale [95, 96]. Our results suggest that Southern hemisphere pandemic risk will be greatest in September and October following their respective flu season [97]. Tropical and subtropical regions, which have low levels of sporadic flu transmission, seasonal patterns, or bimodal seasonality should experience refractory periods in the wakes of their respective epidemics [98–102]. Estimating spatiotemporal emergence risks will require data-driven models that consider local flu seasonality and contact networks, both of which can vary greatly with climatic zone and human developmental index. Such analyses can support pandemic planning, including the targeting of surveillance systems for detecting emergence events around the globe [10, 103].

Our model makes several assumptions about the transmissibility of both seasonal and pandemic influenza viruses. We assume that the intrinsic transmission rates depend on humidity (Fig 5), and do not explicitly consider other environmental and sociological factors that may be important (e.g., school calendar) [33, 104]. Since we estimated pandemic emergence locations and dates based on reports of major outbreaks, our estimates may be biased towards regions with high reporting rates or population densities. Our study is further limited by the small number of pandemic emergence event; with five natural pandemics emerging in subtropical and temperate climates, we lack the power to fit high resolution predictive model. Instead, we used the North American 2008-2009 influenza season as a prototypical flu season for exploring seasonal and immunological drivers of pandemic risk. The flu seasons preceding the other 20th century pandemics likely varied in both timing and magnitude. Additional historical data from those pandemics and their preceding seasons might enable more reliable spatiotemporal estimates of pandemic emergence risk and the duration of cross-immunity.

Recent pandemics exhibited similar timing and geographic origins, having all emerged in the Northern Hemisphere. Why this is so, and whether it suggests higher risk of future pandemic emergence in the Northern Hemisphere is yet unknown. Molecular analyses suggest that seasonal flu diversity is seeded in the Northern Hemisphere (Southeast Asia) [41]. Furthermore, human and livestock populations tend to have higher densities in the Northern Hemisphere than the Southern Hemisphere [105, 106]. These two factors could suggest that the Northern Hemisphere may be a likely source for future pandemics. If influenza refractory periods are estimated for other climatic zones, as we have done here for the Northern Hemisphere, we may better understand the common origins of past pandemics and gain actionable insights into global dynamics of pandemic risk.

We also focus exclusively on transient heterosubtypic immunity immediately following seasonal flu infection, which is only one of many forms of immunological heterogeneity that may constrain pandemic emergence. For example, the age-specific rate of severe and deadly infections of novel H7N9 and H5N1 in China reflect long-lasting heterosubtypic immunity stemming from early childhood influenza infections [48]. Our model does not consider such long-term heterogeneity in susceptibility, nor does it consider intrinsic heterogeneity in heterosubtypic immunity following infection (e.g., variation in severity, transmissibility, or infectious period) [45, 57]. Incorporating historically-acquired immunity, individual heterogeneity, and future advances in our understanding of transient heterosubtypic immunity should improve pandemic risk assessments.

Our study is intended as a proof of concept. Using simple, conservative models of influenza transmission, cross-immunity, and seasonality, we lend support to a parsimonious explanation for the historical spring-summer timing of pandemic emergence and demonstrate that pandemic risk may be both seasonal and predictable. However, there is much we still cannot predict, such as when and where reassorted viruses capable of human-to-human transmission will arise. Recent human outbreaks of H7N9 and H5N1 influenza during the winter and spring months suggest that other factors may inhibit spread, such as intrinsic transmissibility and the underlying immunological landscape [48, 107, 108]. Although we do not address the biogeographic risks of novel viruses first arising through reassortment events in humans or livestock, laboratory experimentation, or other mechanisms, our study provides insight into the subsequent risk of emergence and a method for estimating such risk from seasonal flu surveillance data. As we gain a better understanding of breadth and duration of heterosubtypic immunity, both in general and between specific combinations of influenza viruses, our insights and methodology can be applied to improve global surveillance, detection, planning and intervention efforts for pandemic influenza.

## Supporting information

S1 Fig Two-strain model diagram. Short-term heterosubtypic immunity model description for a single individual (node) in the network. Solid arrows indicate the individual’s transitions through epidemiological states, and dashed arrows indicate neighbor influence on the individual’s transitions, with *n*_*I*_*XY*__ indicating the number of the individual’s neighbors who are currently in state *I*_*XY*_. Symbols labeling arrows indicate the transition rates between states (solid arrows), or the rate at which individuals transmit to susceptible individuals (dashed arrows). For example, an individual in state *S*_21_ has been infected and recovered from disease 2 and is currently susceptible to disease 1, so this individual will become exposed to disease 1 at rate (*n*_*I*_01__ + *n*_*I*_21__)*β*_1_, where *β*_1_ is the per contact rate of transmission for disease 1, and *n*_*I*_01__ + *n*_*I*_21__ is the number of its neighbors who are currently infected with disease 1.
(PDF)

S2 Fig Probability distribution for immune duration. We model the immune duration as an exponentially distributed random variable with rate = 1/42, meaning the most likely immune duration is nearly zero days of immunity, but on average individuals will spend 42 days in the immune state. As the influenza epidemics we model last 100 days or more, the immune duration allows for an individual to experience serial infections of the seasonal and pandemic strain.
(PDF)

S3 Fig The extent and magnitude of the pandemic refractory period depends on the duration of cross-immunity. Columns represent the duration of cross-immunity (*α*), and the rows represent the two networks considered. Lines represent the emergence probability of pandemics across the 2008-2009 seasonal influenza epidemic for a variety of pandemic *R*_0_*s* (colors).
(PDF)

S4 Fig Larger seasonal epidemics produce larger pandemic refractory periods. **A**: Actual 2003-2004 epidemic curve (solid black line) and 200 stochastic simulations of seasonal epidemics for each network (green for empirical; purple for homogeneous), assuming transmission parameters estimated from 2003-2004 data. **B**: The probability of pandemic emergence upon the introduction of a single infected individual, assuming that the pandemic virus has the same transmission rate as the seasonal virus. Probability is estimated as the proportion of introductions that subsequently infected at least 5% of the overall population out of the 5,000 simulations. Horizontal dashed lines indicate the emergence probabilities in a completely susceptible population calculated with Eq 2 from the manuscript. The pandemic refractory periods (shaded regions) are expected to occur during and immediately following the seasonal epidemic peak. **C**: Underestimation of pandemic *R*_0_. Assuming that the emerging pandemic has an *R*_0_ = 3 in a naïve population (dashed horizontal line), we plot the median (points) and interquartile range of the measured *R*_eff_, for each introduction time and each network. For example, if a pandemic with *R*_0_ = 3 emerged in January of 2003 and we did not account for population immunity, we would interpret the *R*_eff_ as the *R*_0_ and considerably underestimate the true transmission rate (*R*_0_ ≈ 2), regardless of our contact network assumptions.
(PDF)

S5 Fig Seasonal flu disconnects the susceptible portion of a population (Large seasonal epidemic). **A**: For a single (typical) 2003-2004 seasonal epidemic simulation, the number of individuals susceptible to infection by a pandemic virus and the number of edges connecting two such individuals are plotted for each network (green for empirical; purple for homogeneous), with each point representing a single time point over the course of the epidemic. Arrows indicate temporal progression. For any given number of remaining susceptible individuals, the empirical model is always sparser than the homogeneous model (that is, it has fewer contacts remaining between susceptibles). **B**: The distribution of degrees (number of contacts) assumed for the empirical model. The homogeneous model assumes that all individuals have 16 contacts. **C** Snapshot of the susceptible portion of the empirical network at the base of the refractory period (at the time point indicated in panel A by the box labeled ‘C’). Points indicate the percent of the nodes that are immune to pandemic infection, across different levels of connectivity. (We bin degrees by 10; for example, the lowest bin includes individuals with 1 to 10 contacts). For comparison, the horizontal dashed line indicates the overall proportion of individuals immunized in the network at the base of the refractory period. In comparison to an individual with an average number of contacts, a highly connected individual will be more vulnerable to seasonal flu infection, and, once infected and immunized, cause greater epidemiological disruption.
(PDF)

S6 Fig Seasonality constrains pandemic emergence timing for the 2003-2004 season. Probability density for pandemic emergence timing for pandemics that emerge during the seasonal influenza epidemic for the homogeneous (purple) and empirical (green) networks. Pandemic emergence timing, the time in which the simulated pandemic begins rapid spread, is defined as the day the pandemic strain incidence reaches five or more cases. Results are for a pandemic emerging during the 2003-2004 flu season with the same transmission rate as the seasonal epidemic. Vertical dashed lines indicate the timing of historic pandemics.
(PDF)

S7 Fig Pandemic emergence timing if pandemics introduced throughout the year (2008-2009). Probability density for pandemic emergence timing for pandemics that emerge across the whole year during and following the seasonal epidemic for the homogeneous (purple) and empirical (green) networks. Pandemic emergence timing, the time in which the simulated pandemic begins rapid spread, is defined as the day the pandemic strain incidence reaches five or more cases. Results are for the 2008-2009 flu season with the same transmission rate as the seasonal epidemic. Vertical lines indicate the timing of historic pandemics, with the solid line indicating the timing of the 2009 pandemic and dashed lines indicating timing of others.
(PDF)

S8 Fig Pandemic emergence if pandemics introduced throughout the year (2003-2004). Probability density for pandemic emergence timing for pandemics that emerge across the whole year during and following the seasonal epidemic for the homogeneous (purple) and empirical (green) networks. Pandemic emergence timing, the time in which the simulated pandemic begins rapid spread, is defined as the day the pandemic strain incidence reaches five or more cases. Results are for the 2003-2004 flu season with the same transmission rate as the seasonal epidemic. Vertical dashed lines indicate the timing of historic pandemics.
(PDF)

S9 Fig Pandemic refractory period reduces as transmissibility increases. Pandemic emergence probabilities plotted for the 2008-2009 seasonal simulation for a pandemic that is less transmissible than the seasonal strain (Top) and one that is more transmissible than the seasonal strain (Bottom) on the two analyzed networks (fill colors). Probability is estimated as the proportion of introductions that subsequently infected at least 5% of the overall population out of the 5,000 simulations. Horizontal dashed lines indicate the emergence probabilities in a completely susceptible population calculated with Eq 2. The pandemic refractory periods (shaded regions) are plotted the same as in the manuscript. Refractory period is deeper and wider for the less transmissible strain, and nearly disappears if the pandemic transmissibility is high enough.
(PDF)
