# Supplementary material for: Seasonality in risk of pandemic influenza emergence
Source: PLoS Comput Biol. 2017 Oct 19;13(10):e1005749. doi: 10.1371/journal.pcbi.1005749 (PMC5654262; doi:10.1371/journal.pcbi.1005749)

Parameters

β_1	Disease 1 per edge transmission rate
β_2	Disease 2 per edge transmission rate
η	1/incubation period = 1/3.38 days
γ	1/infectious period = 1/2.26 days
α	1/protected period = 1/42 days

Individual States

S	Fully Susceptible	S_{12}	Susceptible d2 post d1
E_{01}	Exposed disease 1	S_{21}	Susceptible d1 post d2
E_{02}	Exposed disease 2	E_{12}	Exposed d2 post d1
I_{01}	Infectious disease 1	E_{21}	Exposed d1 post d2
I_{02}	Infectious disease 2	I_{12}	Infectious d2 post d1
P_1	Protected	I_{21}	Infectious d1 post d2
P_2	Protected	R	Fully Recovered

$$N = 10{,}000 = S+E_{01}+E_{02}+I_{01}+I_{02}+P_1+P_2+S_{12}+S_{21}+E_{12}+E_{21}+I_{12}+I_{21}+R$$

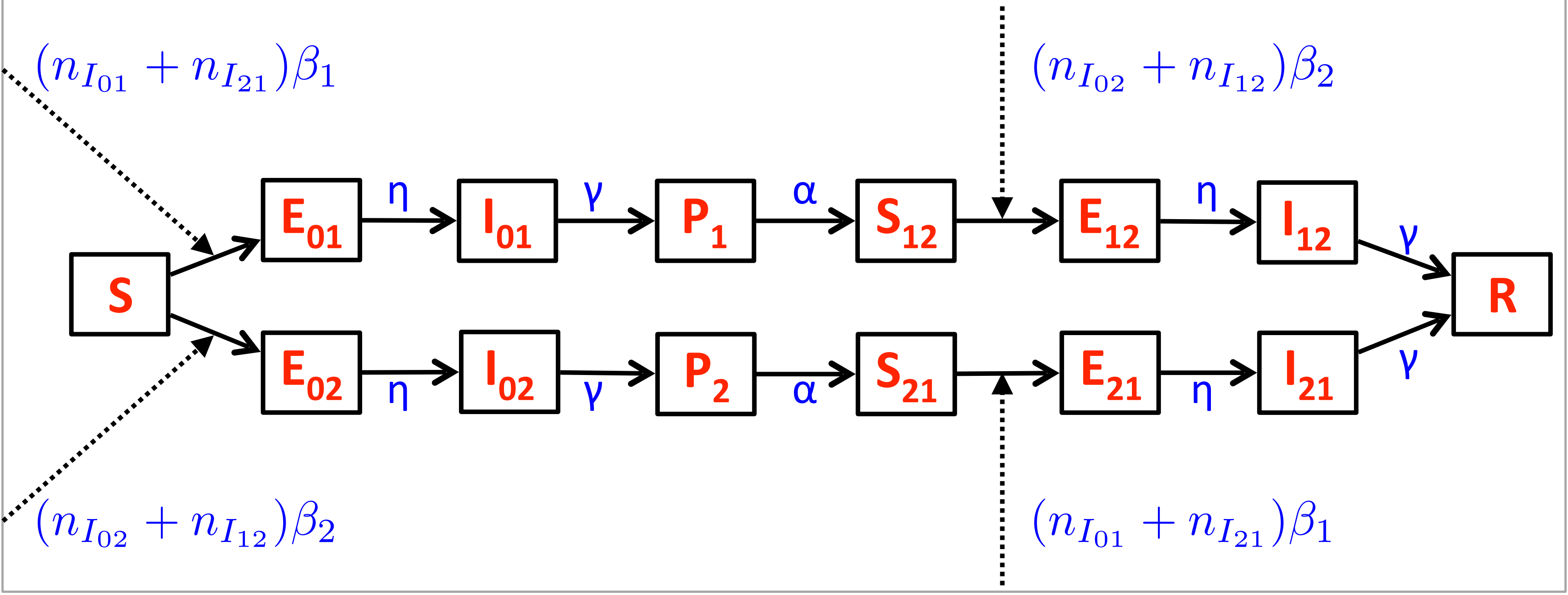

Supplement: S1 Fig — Short-term heterosubtypic immunity model description for a single individual (node) in the network. Solid arrows indicate the individual’s transitions through epidemiological states, and dashed arrows indicate neighbor influence on the individual’s transitions, with nIXY indicating the number of the individual’s neighbors who are currently in state IXY. Symbols labeling arrows indicate the transition rates between states (solid arrows), or the rate at which individuals transmit to susceptible individuals (dashed arrows). For example, an individual in state S21 has been infected and recovered from disease 2 and is currently susceptible to disease 1, so this individual will become exposed to disease 1 at rate (nI01 + nI21)β1, where β1 is the per contact rate of transmission for disease 1, and nI01 + nI21 is the number of its neighbors who are currently infected with disease 1. (PDF) [file pcbi.1005749.s001.pdf]
