# Supplementary figures and images for: Seasonality in risk of pandemic influenza emergence
Source: PLoS Comput Biol. 2017 Oct 19;13(10):e1005749. doi: 10.1371/journal.pcbi.1005749 (PMC5654262; doi:10.1371/journal.pcbi.1005749)

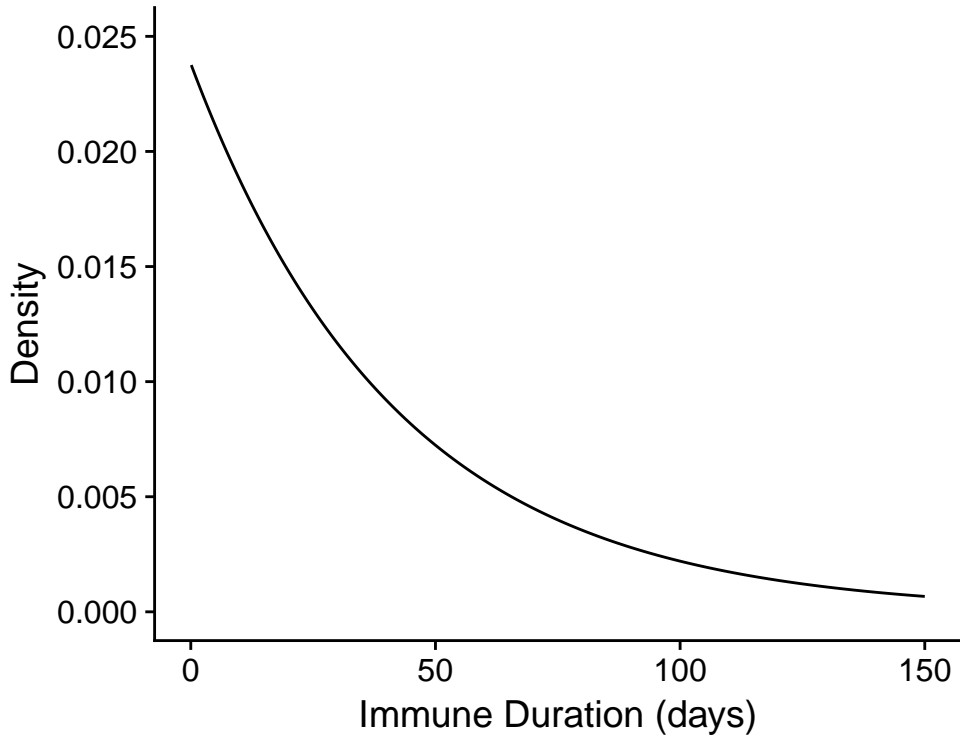

Supplement: S2 Fig — We model the immune duration as an exponentially distributed random variable with rate = 1/42, meaning the most likely immune duration is nearly zero days of immunity, but on average individuals will spend 42 days in the immune state. As the influenza epidemics we model last 100 days or more, the immune duration allows for an individual to experience serial infections of the seasonal and pandemic strain. (PDF) [file pcbi.1005749.s002.pdf]

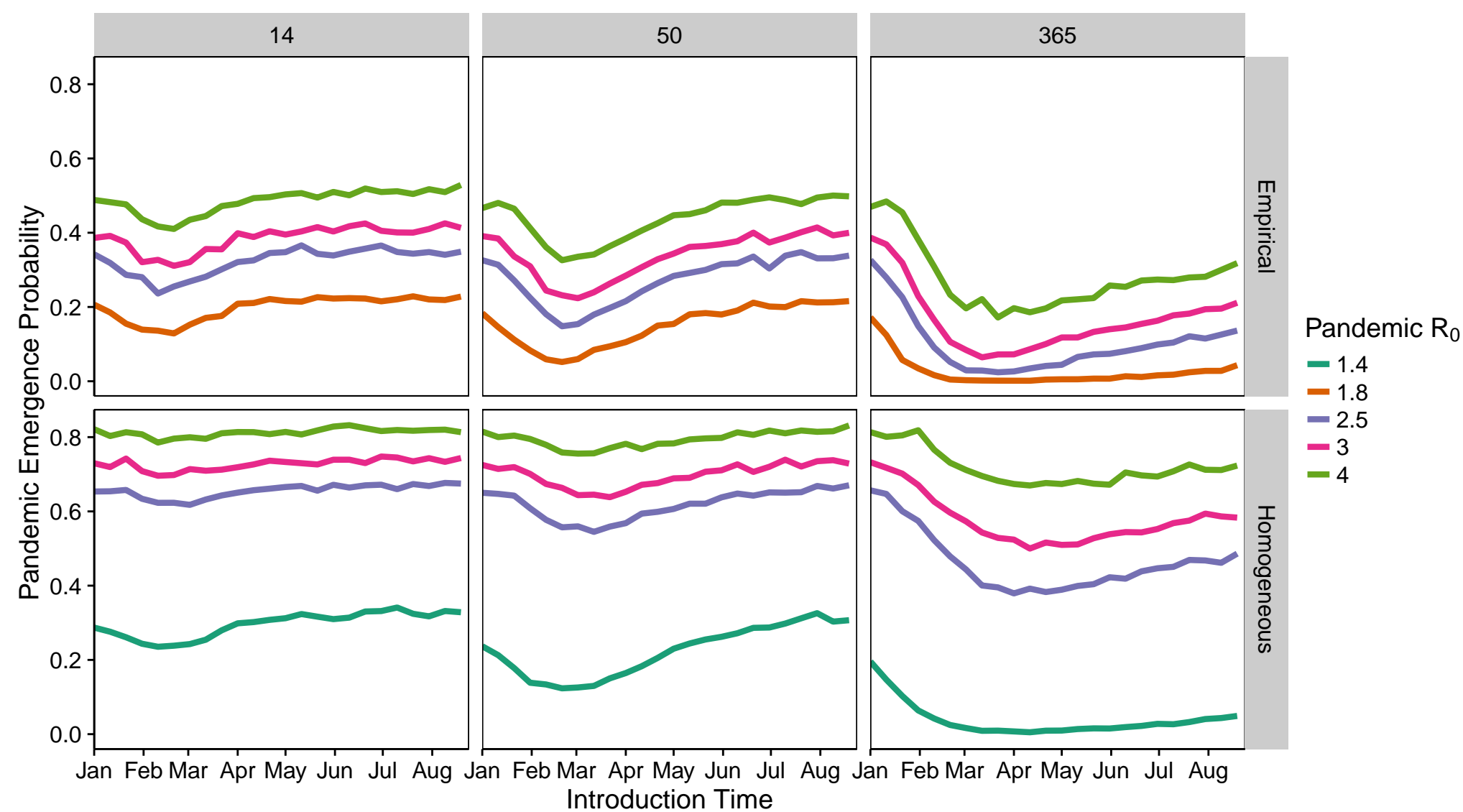

Supplement: S3 Fig — Columns represent the duration of cross-immunity (α), and the rows represent the two networks considered. Lines represent the emergence probability of pandemics across the 2008-2009 seasonal influenza epidemic for a variety of pandemic R0s (colors). (PDF) [file pcbi.1005749.s003.pdf]

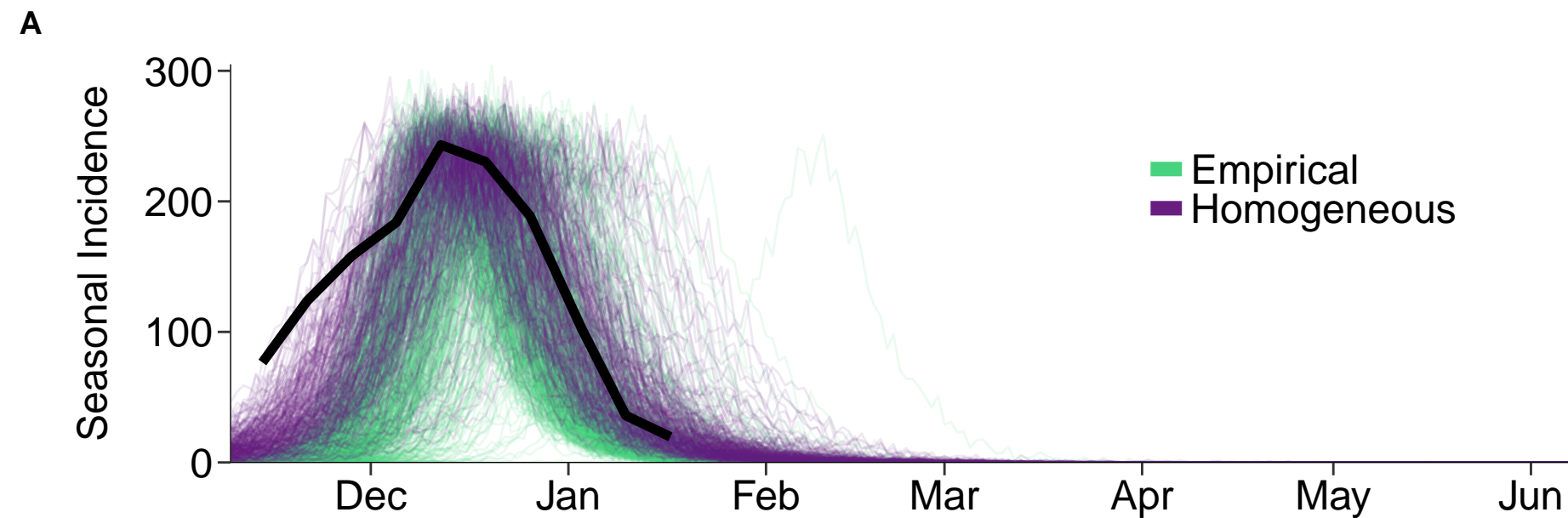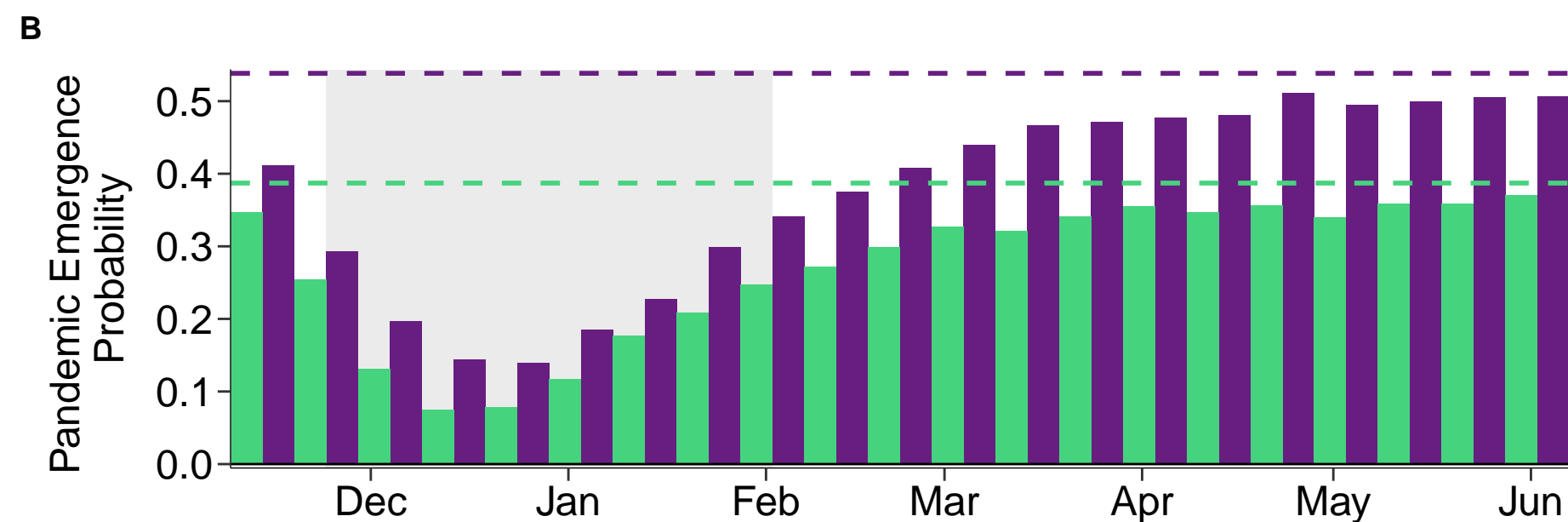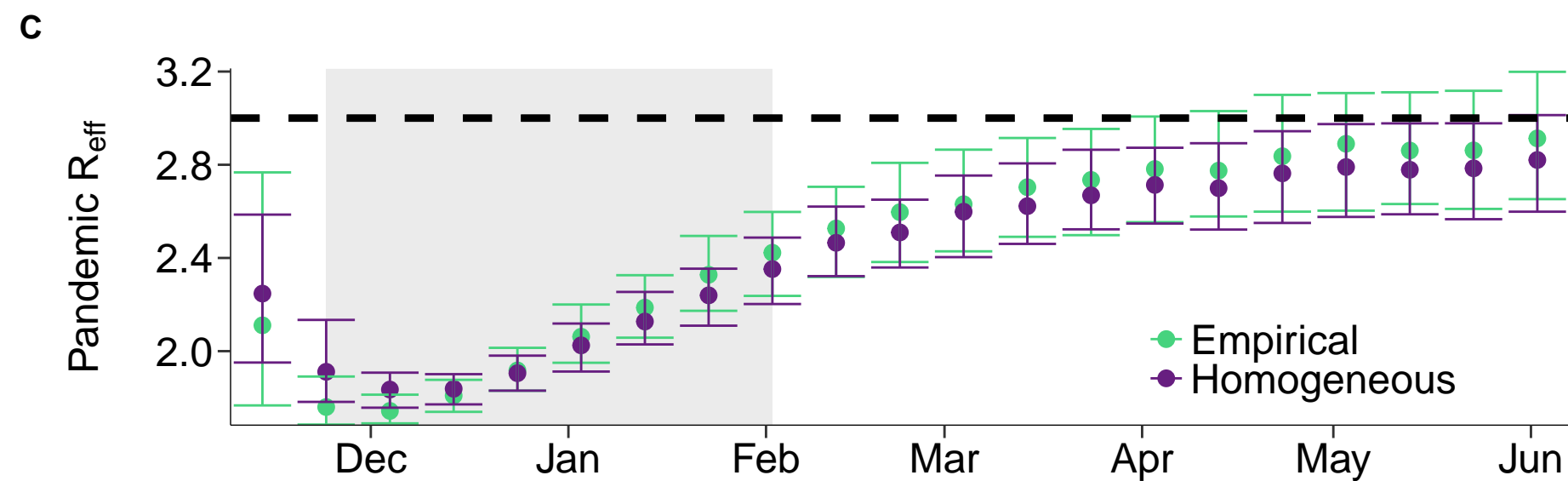

Supplement: S4 Fig — A: Actual 2003-2004 epidemic curve (solid black line) and 200 stochastic simulations of seasonal epidemics for each network (green for empirical; purple for homogeneous), assuming transmission parameters estimated from 2003-2004 data. B: The probability of pandemic emergence upon the introduction of a single infected individual, assuming that the pandemic virus has the same transmission rate as the seasonal virus. Probability is estimated as the proportion of introductions that subsequently infected at least 5% of the overall population out of the 5,000 simulations. Horizontal dashed lines indicate the emergence probabilities in a completely susceptible population calculated with Eq 2 from the manuscript. The pandemic refractory periods (shaded regions) are expected to occur during and immediately following the seasonal epidemic peak. C: Underestimation of pandemic R0. Assuming that the emerging pandemic has an R0 = 3 in a naïve population (dashed horizontal line), we plot the median (points) and interquartile range of the measured Reff, for each introduction time and each network. For example, if a pandemic with R0 = 3 emerged in January of 2003 and we did not account for population immunity, we would interpret the Reff as the R0 and considerably underestimate the true transmission rate (R0 ≈ 2), regardless of our contact network assumptions. (PDF) [file pcbi.1005749.s004.pdf]

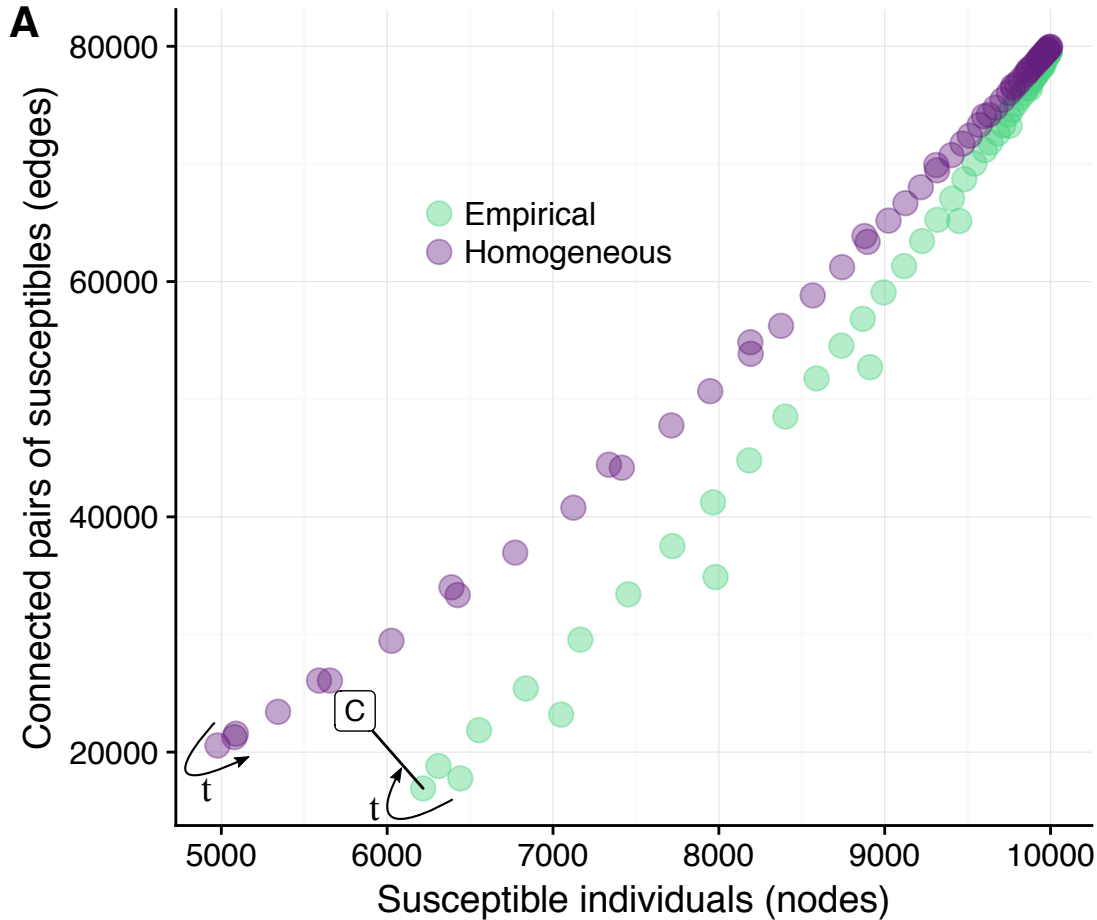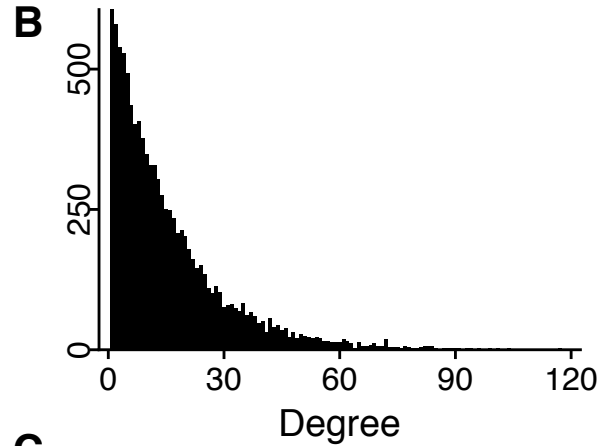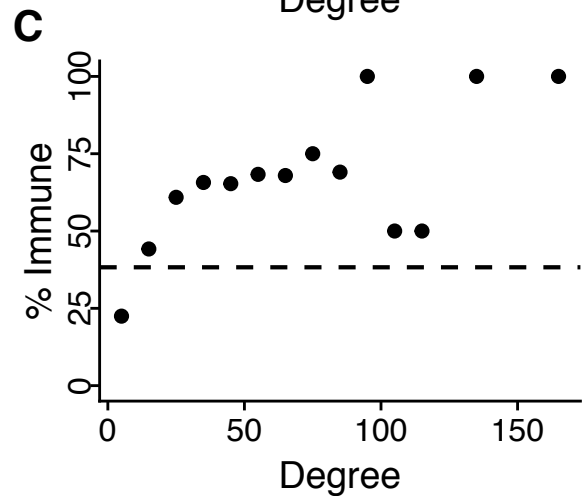

Supplement: S5 Fig — A: For a single (typical) 2003-2004 seasonal epidemic simulation, the number of individuals susceptible to infection by a pandemic virus and the number of edges connecting two such individuals are plotted for each network (green for empirical; purple for homogeneous), with each point representing a single time point over the course of the epidemic. Arrows indicate temporal progression. For any given number of remaining susceptible individuals, the empirical model is always sparser than the homogeneous model (that is, it has fewer contacts remaining between susceptibles). B: The distribution of degrees (number of contacts) assumed for the empirical model. The homogeneous model assumes that all individuals have 16 contacts. C Snapshot of the susceptible portion of the empirical network at the base of the refractory period (at the time point indicated in panel A by the box labeled ‘C’). Points indicate the percent of the nodes that are immune to pandemic infection, across different levels of connectivity. (We bin degrees by 10; for example, the lowest bin includes individuals with 1 to 10 contacts). For comparison, the horizontal dashed line indicates the overall proportion of individuals immunized in the network at the base of the refractory period. In comparison to an individual with an average number of contacts, a highly connected individual will be more vulnerable to seasonal flu infection, and, once infected and immunized, cause greater epidemiological disruption. (PDF) [file pcbi.1005749.s005.pdf]

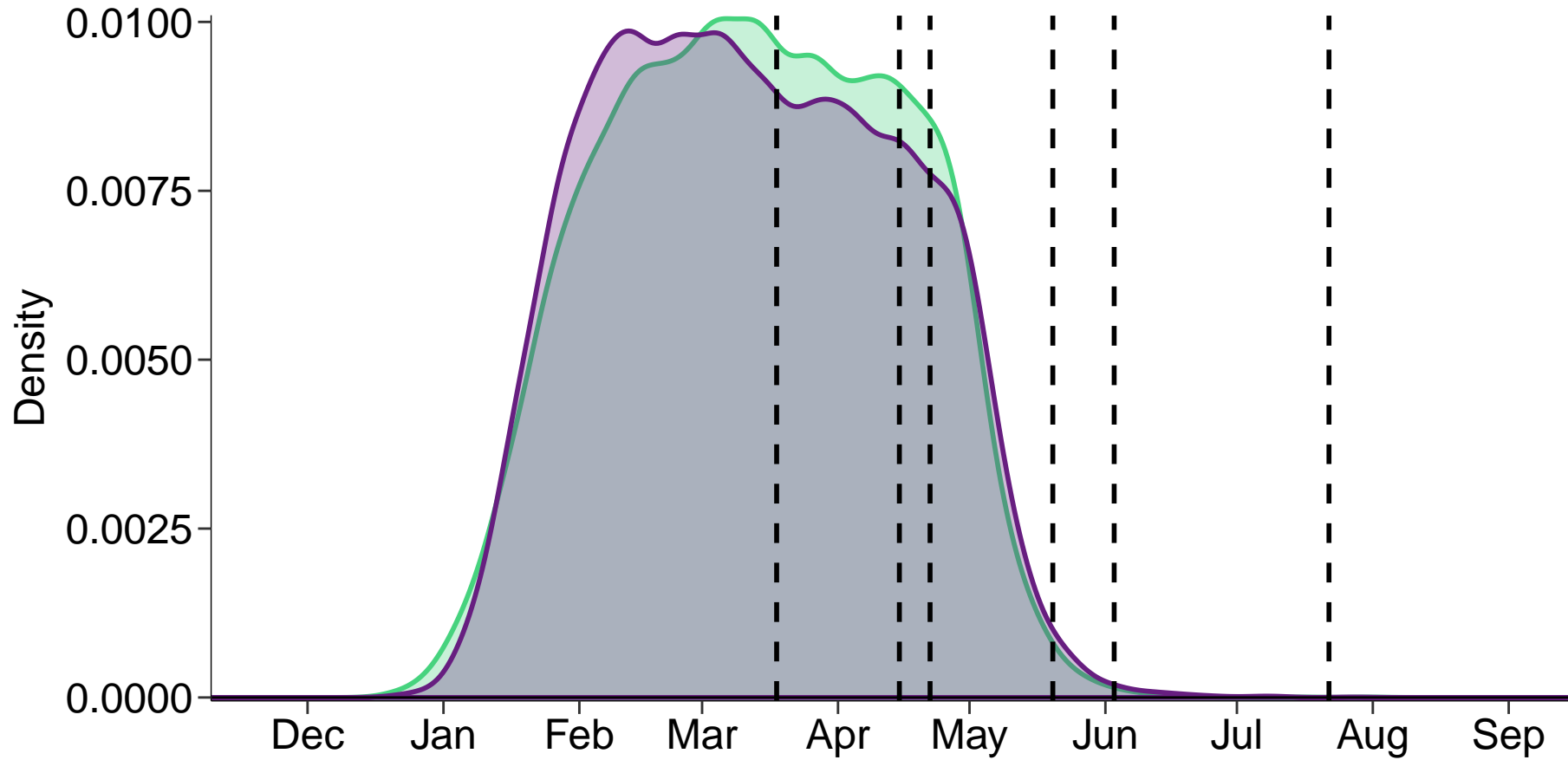

Supplement: S6 Fig — Probability density for pandemic emergence timing for pandemics that emerge during the seasonal influenza epidemic for the homogeneous (purple) and empirical (green) networks. Pandemic emergence timing, the time in which the simulated pandemic begins rapid spread, is defined as the day the pandemic strain incidence reaches five or more cases. Results are for a pandemic emerging during the 2003-2004 flu season with the same transmission rate as the seasonal epidemic. Vertical dashed lines indicate the timing of historic pandemics. (PDF) [file pcbi.1005749.s006.pdf]

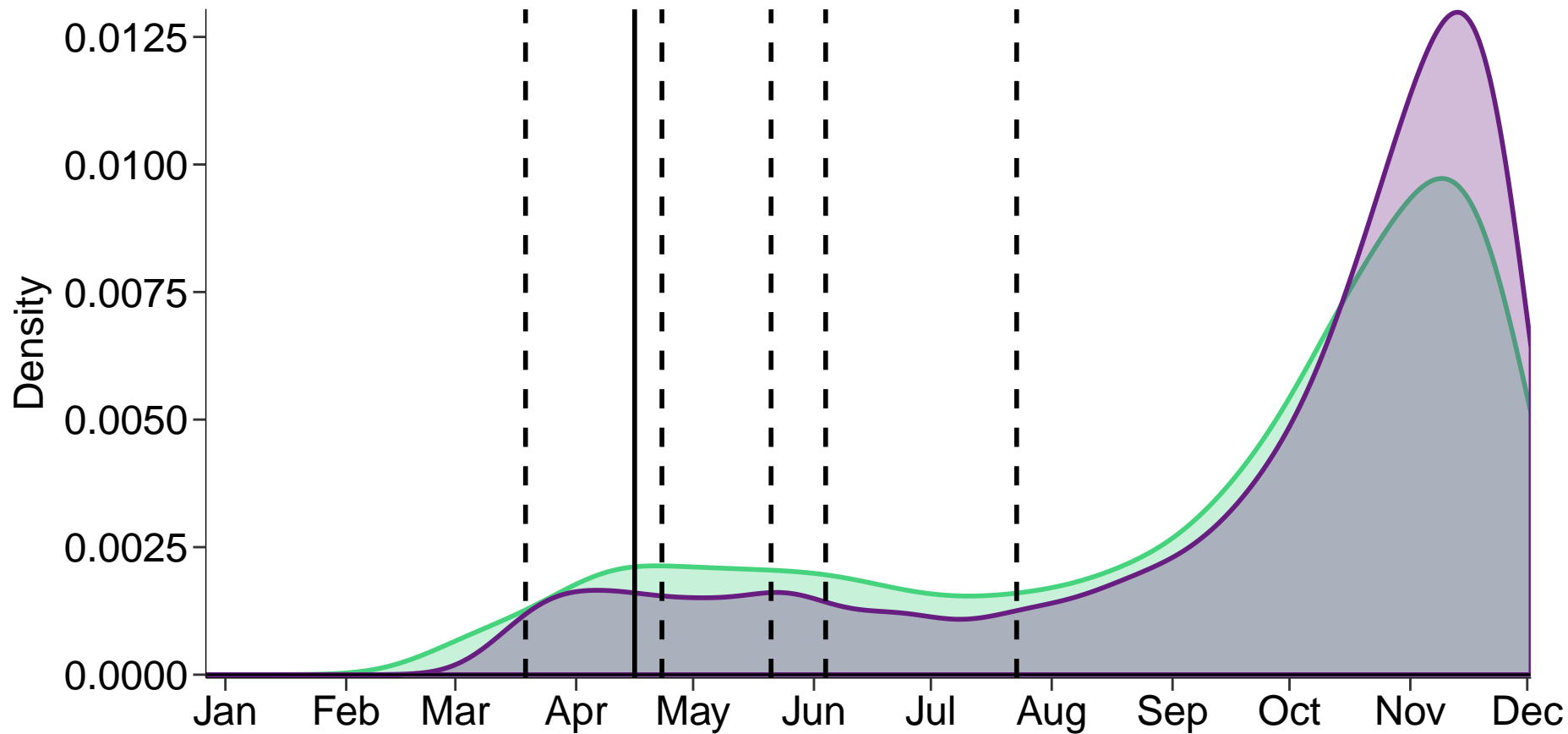

Supplement: S7 Fig — Probability density for pandemic emergence timing for pandemics that emerge across the whole year during and following the seasonal epidemic for the homogeneous (purple) and empirical (green) networks. Pandemic emergence timing, the time in which the simulated pandemic begins rapid spread, is defined as the day the pandemic strain incidence reaches five or more cases. Results are for the 2008-2009 flu season with the same transmission rate as the seasonal epidemic. Vertical lines indicate the timing of historic pandemics, with the solid line indicating the timing of the 2009 pandemic and dashed lines indicating timing of others. (PDF) [file pcbi.1005749.s007.pdf]

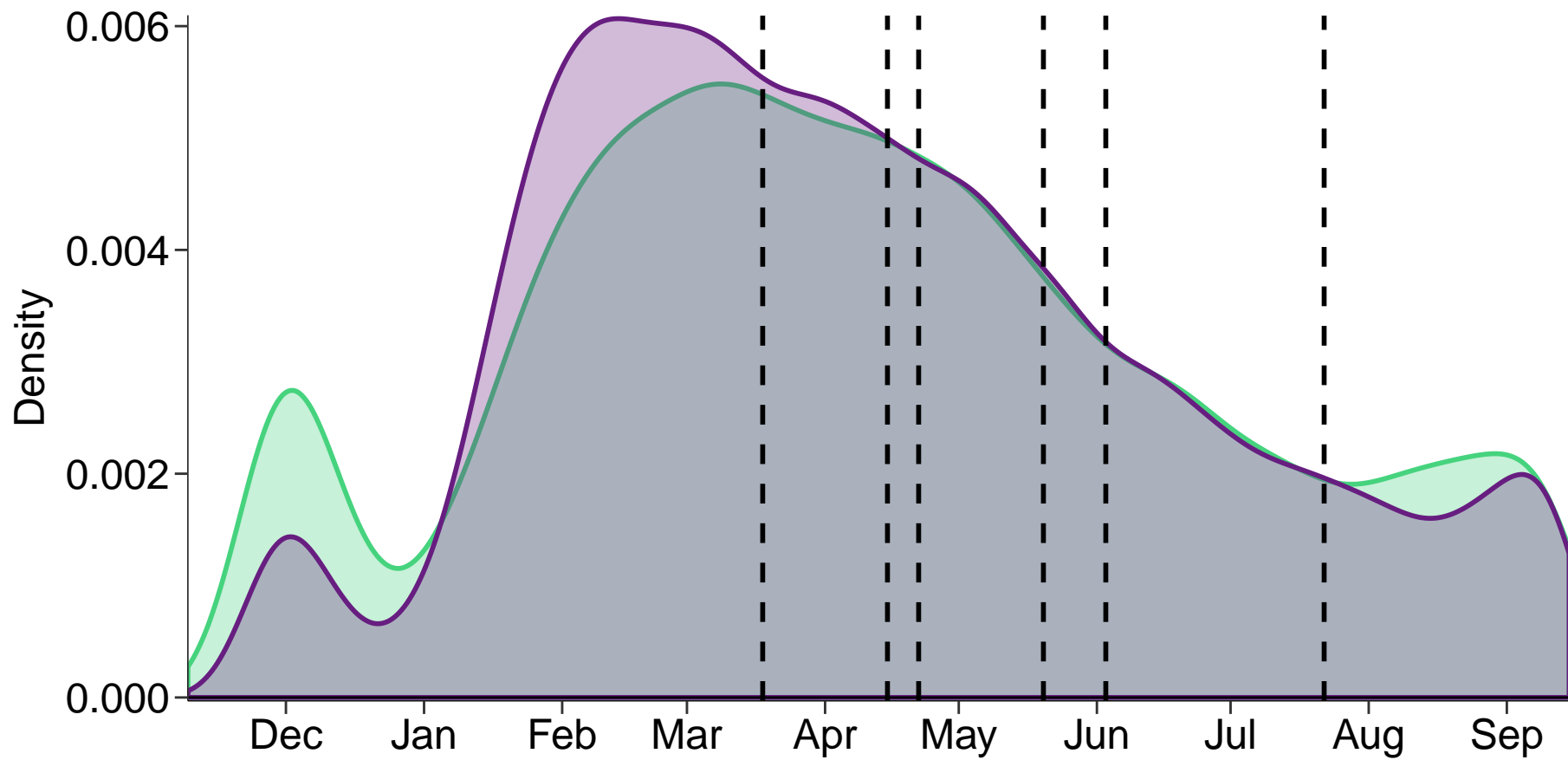

Supplement: S8 Fig — Probability density for pandemic emergence timing for pandemics that emerge across the whole year during and following the seasonal epidemic for the homogeneous (purple) and empirical (green) networks. Pandemic emergence timing, the time in which the simulated pandemic begins rapid spread, is defined as the day the pandemic strain incidence reaches five or more cases. Results are for the 2003-2004 flu season with the same transmission rate as the seasonal epidemic. Vertical dashed lines indicate the timing of historic pandemics. (PDF) [file pcbi.1005749.s008.pdf]

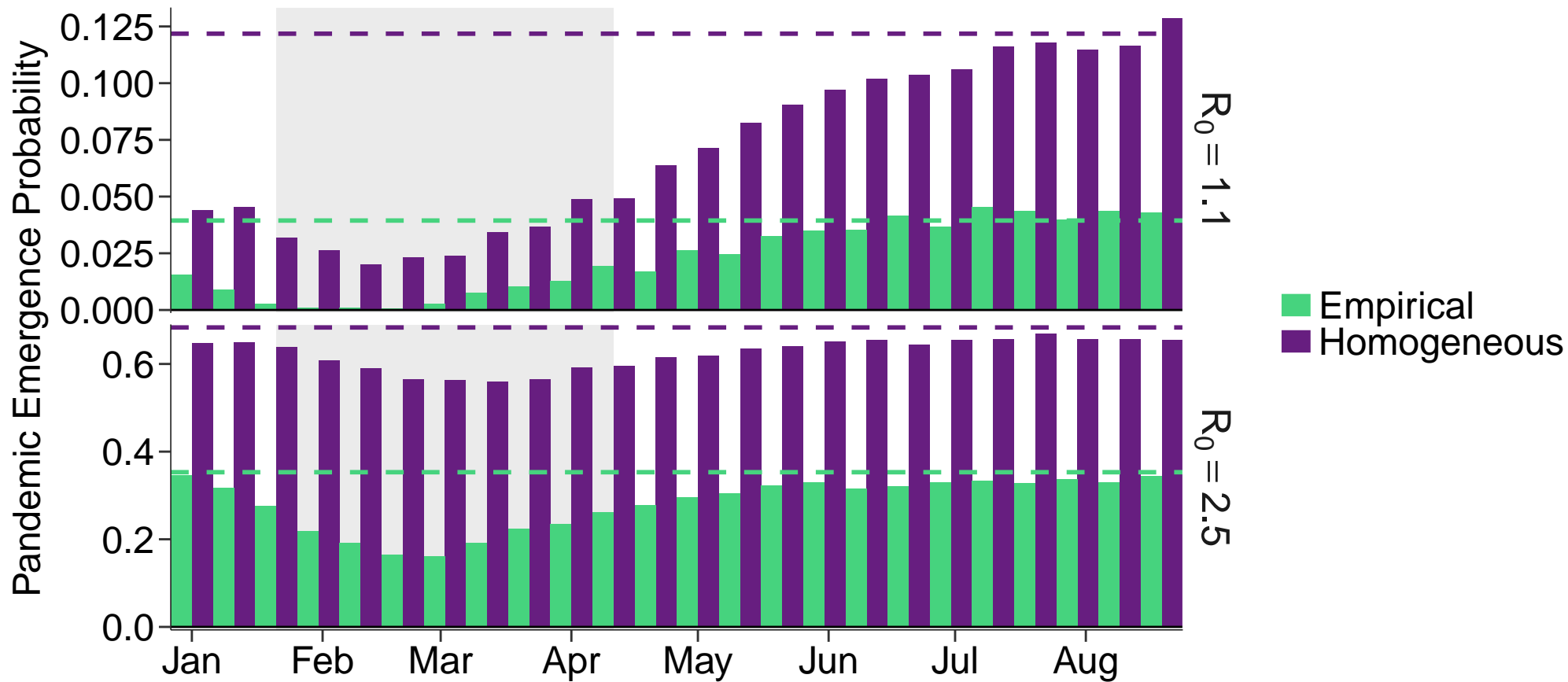

Supplement: S9 Fig — Pandemic emergence probabilities plotted for the 2008-2009 seasonal simulation for a pandemic that is less transmissible than the seasonal strain (Top) and one that is more transmissible than the seasonal strain (Bottom) on the two analyzed networks (fill colors). Probability is estimated as the proportion of introductions that subsequently infected at least 5% of the overall population out of the 5,000 simulations. Horizontal dashed lines indicate the emergence probabilities in a completely susceptible population calculated with Eq 2. The pandemic refractory periods (shaded regions) are plotted the same as in the manuscript. Refractory period is deeper and wider for the less transmissible strain, and nearly disappears if the pandemic transmissibility is high enough. (PDF) [file pcbi.1005749.s009.pdf]
